# Thermal shock treatment of recyclable bimetallic MOF derived carbon composite for organics oxidation by advanced Fenton-Like technique

**DOI:** 10.1038/s41598-025-13124-x

**Published:** 2025-08-12

**Authors:** Safa H. Monir, Osama Abuzalat, Ibrahim E.T. El-Sayed, Hamed M. Abdel-Bary, Maha A. Tony

**Affiliations:** 1 Advanced Materials/Solar Energy and Environmental Sustainability (AMSEES) Laboratory, Basic Engineering Science Department, Faculty of Engineering, Menoufia University, Shebin El-Kom, Egypt; 2 Chemistry department, Faculty of Science, Menoufia University, Shebin El- Kom, Egypt; 3 Department of Chemical Engineering, Military Technical College, Cairo, Egypt; 4 Planning & Construction of Smart Cities Program, Faculty of Engineering, Menoufia National University, Menoufia, 32651 Egypt

**Keywords:** MOF, Carbon, Catalysis, Fenton, Dye discharge, Pharmaceutical effluents, Environmental sciences, Environmental chemistry

## Abstract

This study offers a bimetallic MIL-88 B Metal–Organic Frameworks (MOF) derived carbon composite Co/Ferrite MOF namely Co/Fe@C that is synthesized through a solvothermal route followed by a simple thermal shock treatment and used as a Fenton-like source. The synthesized Co/Fe@C morphology and elemental analysis are characterized via X-ray diffraction (XRD), Fourier transform infrared spectroscopy (FTIR), and field emission scanning electron microscopy (FE-SEM), X-ray photoelectron spectroscopy (XPS), Brunauer–Emmett–Teller (BET) and vibrating sample magnetometer (VSM). Carbon based MOF demonstrated fascinating features as a Fenton source conducted in dark oxidation route. The material exposed a superior efficiency in treating various organic pollutants including basic (Malachite Green, MG) and acidic (Oil Orange SS, OOSS) dyes as a textile simulated effluent and tetracycline (TC) as a model pharmaceutical wastewater. The experimental results exhibited the optimum reaction conditions of 400 mg/L for H_2_O_2_ for all contaminants oxidation and ranged from 10 to 40 mg/L for Co/Fe@C catalyst at varied optimal pH values. Under optimal conditions, the Co/Fe@C catalyst achieved removal efficiencies reached to 100% for MG, 83% for OOSS, and 72% for TC within 30 min. Furthermore, for potential full-scale application, the kinetic investigation is highlighted and the reaction is following the second kinetic order. Also, to assure catalyst sustainability, the Co/Fe@C substance is reused after recovery for seven oxidation cycles with a reasonable decline in its activity that reached to 57, 45 and 42% removals for MG, OOSS and TC, respectively. Furthermore, the mechanism exploration indicated the active species involved oxidation process primarily affecting TC and OOSS oxidation is holes (h^+^) and MG is OH radicals.

## Introduction

Humanity is facing from inescapable environmental contamination that is posed a threat to human health and ecosystem. Additionally, modern societies are signified with industrialization, which consumes massive amounts of freshwater and thereby the result is high amount of disposed toxic contaminants embedded wastewater that causing a threat to the water resources^1,2^. Nowadays, a variety of industrial discharge such as dyes and pharmaceuticals effluent are detected in aquatic environment thus, the industrial pollution is getting worse^3^. Thus, such discharge once is released into the watercourse are signified as toxic for both human health and environmental system^4^. In this regard, this deterioration for the ecosystem must be treated. But, due to the stable chemical structure of those substances, their treatment is still struggling both academia and industrial sector^5^.

Conventional physical, chemical and biological wastewater treatments could be signified as feasible wastewater treatment opportunity^6^. Among such affordable techniques filtration, coagulation^7^, precipitation^8^, ion exchange^9^, bio treatments^10^, adsorption^11^ and advanced oxidations processes^12^ are available. The application of such existing technologies in wastewater treatment is still require an improvement to make them more economic and reliable in application in order to meet water quality criteria. Although chemical treatments among the available facilities is signified as viable treatment opportunity, it possess some demerits including high operational and chemical costs besides final sludge formation after treatments requires further treatment that is a problematic topic^13^. From this regard, urgent search for new technologies using environmentally benign materials to eliminate such toxic substances from aqueous streams is essential. One of the environmentally friendly technologies is advanced oxidation process (AOP) due to its simple operation, mild reaction circumstances and cost efficient^14,15^. Fenton system is one of the AOPs that is signified with its high efficiency and harmless end products^16^. However, conventional homogenous Fenton systems possess some shortcoming including the unrecyclable material and some of the intrinsic disadvantages. Thus, transforming into heterogeneous Fenton catalysts is the solution to address such limitations^17^.

Considerable attention has been received for Metal–Organic Frameworks (MOFs) especially their carbon composite derivatives as great multipurpose materials especially in the field of wastewater treatment. The available metal oxides and other transition metal-doped composite could be applied as a viable heterogeneous Fenton system. But, the narrow working pH range, poor catalytic activity, lesser active sites and insignificant recyclability stands on real applications of such novel heterogeneous substances. Furthermore, conventional Fenton’s system should be initiated by ultraviolet irradiance, ultrasound or microwave radiation, which makes the process costive. Thereby, abundant routs have been developed in advancing heterogeneous Fenton catalysts especially on Fenton based MOF derived substances. MOFs are signified as porous^18^ and crystalline materials^19^ with brilliant number of functional groups^20^. Such amazing features making them gain a considerable attention for the utilization in the area of wastewater treatments^21–23^. However, for more flexibility in developing and designing nanostructured materials, including metal nanoparticles, porous carbons, and the thermal conversion MOFs of are simultaneously deserving attention.

Carbon-based substances developed from MOFs carbonization have been reported with potential benefits including high surface area and inhibits the MOF particles aggregation^24^. Additionally, Fe-MOF was pyrolyzed to create magnetic carbon nanocomposites^25^. Furthermore, MOFs carbonization exposes extra active sites and a lower density compared to the conventional MOF. It is worthily to mention that hollow porous carbon presents in the carbonized MOF improves the cavities in the material and thereby increasing its activity. However, the rationally designed carbon hybrids with elaborate nanostructures with facile fabrication processes are still rarely reported. Also, attaining carbon nanocomposites using MOF precursors entails specific conditions such as pyrolysis in a gaseous environment by applying various pyrolysis gas (i.e. air, O_2_, H_2_, NH_3_, AR or N_2_) and certain high temperatures for long duration of time^26^. Thereby, the process is categorized as energy-intensive and the practical application is a challenge with improving the functional properties and structural features. Hence, significant efforts in synthesis of MOF derived carbon nanocomposite are required^27^. Application of such carbonized MOF as a source of Fenton catalyst might overcome the catalyst agglomeration that could reduce their catalytic activity. Moreover, magnetic recoverable iron MOF containing carbon composites could activate peroxide with high catalytic activity^28^. This is due to the carbonaceous substances possess high conduct electricity, specific surface area and porosity. Such materials can enhance electron transfer and upsurge the active sites for contaminants degradation in water with the advantage of easily catalyst separation^29,30^. Thus, those carbonaceous substances have been widely selected as matrices for loading of magnetic nanoparticles. Thus their combination in Fenton’s oxidation system is superior^31^.

Till now, according to the literature cited there has been a lack of such information to the best of the authors’ knowledge in presenting the activity of a carbonized bimetallic MOF (Co/Fe@C) prepared in mild conditions and introduced as Fenton oxidation system. Herein, the novel porous magnetic carbon composites with iron-based MOF were prepared as a bimetallic Co-Ferrite metal organic framework (MOF) in its carbonized form namely Co/Fe@C using a thermal shock technique. The material synthesis and characterization are presented in details. The catalytic activity of obtained magnetic products-based MOF is also investigated by oxidizing dyes and pharmaceutical effluents through Fenton treatments. Different system variables are assessed and the decisive variable is highlighted. Also, the kinetic modeling is presented as well.

## Experimental investigation

### Synthesis of MIL 88 B MOF and MOF-derived carbon (Co/Fe@C composite)

Iron chloride (FeCl_3_.6H_2_O, 98%), cobalt chloride hexahydrate (CoCl_2_.6H_2_O, 99%), 1,4-benzene dicarboxylic acid (H_2_BDC, 98%), N, N-dimethylformamide (DMF, 98%) and absolute ethanol (ETOH) all are supplied by Sigma Aldrich (USA) and used for the synthesis of (Fe/Co)-BDC. Diluted solutions of hydrochloric acid (HCl, 0.1 N) and sodium hydroxide (NaOH, 0.1 N) (all supplied by Alfa Aesar) were used for the propose of washing the produced material as well as pH adjustment and regeneration purposes. Furthermore, NaCl was also used for calculating the zero-point charge (supplied by Sigma Aldrich (USA).

For aqueous wastewater solution preparation, malachite green (MG), oil orange SS dye (OOSS) supplied by DyStar Ltd., Germany or tetracycline (TC) supplied by Sigma Aldrich (USA) were used. Distilled water was used to prepare such dye solutions. Also, various scavengers were added to the catalytic reaction system in order to investigate the most oxidizing species. Thus, silver nitrate (AgNO_3_), isopropanol (IPA) and ammonium oxalate (AO) are used to trap and remove active species including holes (h^+^), hydroxyl radical (^•^OH radicals), and superoxide radical (^•^O_2¯_), respectively. All the used reagents and chemicals are of analytical grade and used as received without further purification or treatment.

A pristine sample of MIL-88B (Fe/Co) was synthesized following earlier reported data in literature with slight modifications^32^. Initially, 1,4-benzene dicarboxylic acid (H_2_BDC) (0.166 g, 1 mmol), FeCl_3_.6H_2_O (0.135 g, 0.5 mmol) and CoCl_2_.6H_2_O (0.115 g, 0.5 mmol) were poured into a vessel and subjected for stirring through 30 min of reaction time at room temperature through a successive addition. Subsequently, N, N-dimethylformamide (DMF) (30 mL) was poured into the mixture to achieve a complete dissolution. The mixture was then placed in an autoclave and maintained for twenty-four hours at 100 °C. then, the resultant brown powder was recovered by centrifugation at 6000 rpm for 15 min. Afterwards, the resultant material is recovered and subjected for three times repetitive washing with ethanol and DMF prior drying using 10 mL of each per wash.

Thermal shock of MOF is superior due to the ultra-fast reaction rates that occur at high temperature in comparison to the traditional heating methods. Traditional carbonization in an inert atmosphere could yield mixed metal oxides of Fe and Co nanoparticles such as FeO, Fe_3_O_4_ and CoO embedded in carbon. However, to attain a cobalt ferrite composite (CoFe_2_O_4_) without such intermediates, a subsequent oxidation step via calcination in air at high temperature is essential. Moreover, thermal shock technique in air allows for the direct formation of the desired cobalt ferrite and preventing the formation of undesired intermediate phases or less active mixed metal oxides. Also, thermal shock facility successfully stopped the sintering of Fe/Co nanoparticles and avoids their high dispersion on carbon that many cause defects. Furthermore, such rapid thermal decomposition can partially preserve original morphological features that may not be attained with slow pyrolysis offering structural stability and enhanced accessibility to active sites. In an economic manner such single step of calcination simplifying the synthesis procedure whereas no inert gas needed with a reduced calcination time^33^.

**Fig. 1:**
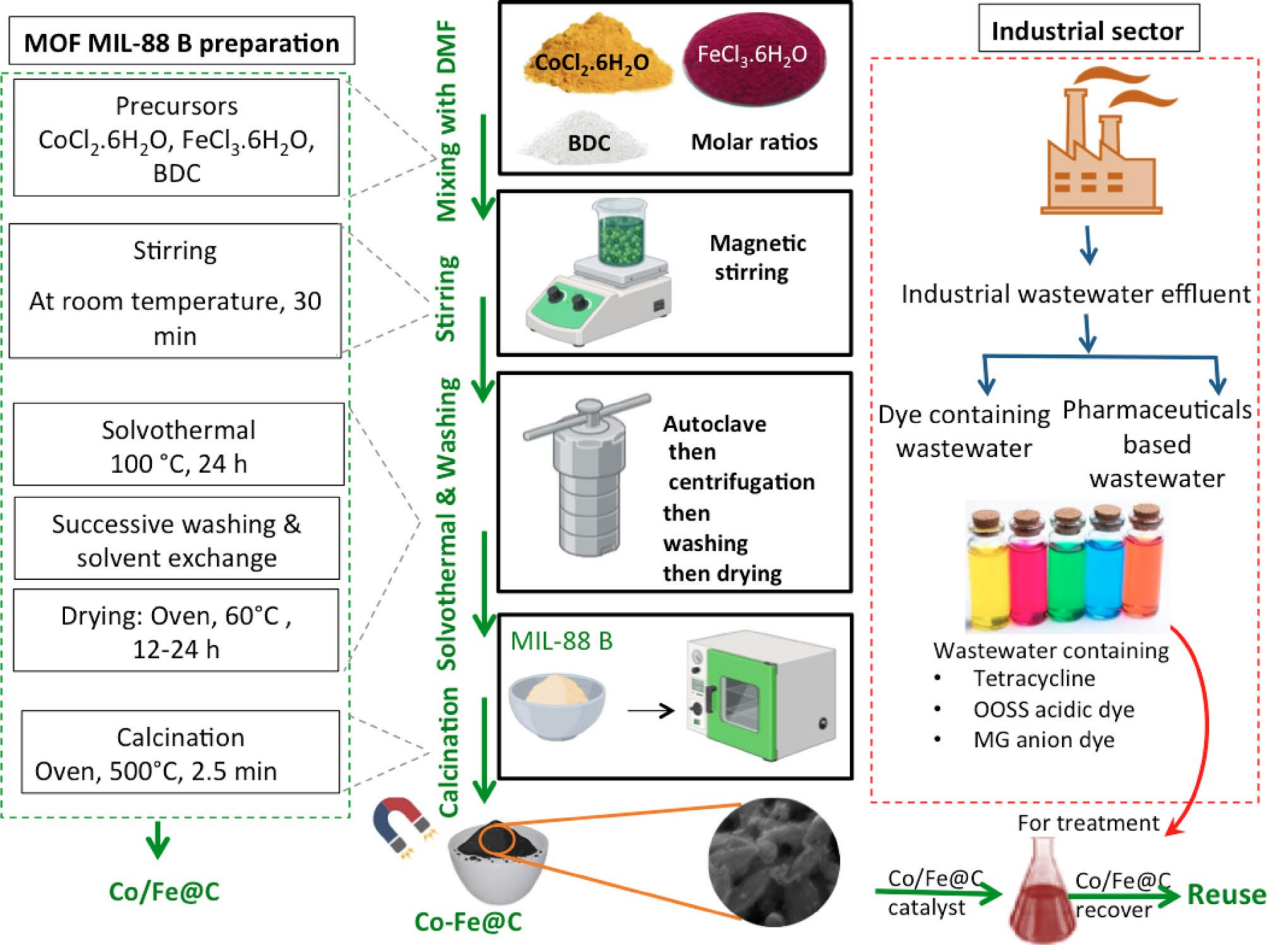
Schematic illustration of experimental setup

Then, the resultant material is exposed to overnight drying at 60 °C followed by vacuum drying at 150 °C for 24 h. Next, the attained material is exposed to carbonization stage based on thermal shock treatment (500 °C for 2.5 min) and Co/Fe@C was finally attained as a black powder and used as a catalytic based material for wastewater treatment. Schematic representation of the preparation and experimental steps is displayed in Fig. 1.

### Fe/Co@C catalyst characterizations

The prepared Fe/Co@C was characterized by a various techniques including: (i) FTIR (KBr pellet method using a NicoLET iS10 FT/IR) model (Bruker Corporation, Billerica, MA, USA) for chemical structure, (ii) powder X-ray diffraction (PXRD, D8-Find, Bruker, with CuKα radiation (1.5418 Å), Madison, WI, USA) working at a current of 40 MA, voltage of 40 kV, and step filter of 0.01º for crystalline nature, (iii) SEM (Quanta FEG 250, FEI Company, Hillsboro, Oregon, USA), (iv) The X-ray photoelectron spectrum (XPS) was acquired on K-ALPHA (Thermo Fisher Scientific, USA) with a monochromatic Al-Kα (−10 to 1350 eV) spot size of 400 micrometers at a pressure of 10 − 9 mbar with full spectrum pass energy of 200 eV and at narrow spectrum 50 eV, (v) BET analysis (N_2_ adsorption, Quanta chrome model of NOVA touch 2LX) for surface area determination and (vi) VSM (Vibrating sample magnetometer VSM lackshore 7410 USA).

### Experimental methodology and analytical determination

The catalytic performance of Co/Fe@C was evaluated for the oxidation removal of both pharmaceutical (TC) and dye (OOSS and MG) effluent. The dyes provided by DyStar Ltd., Germany and utilized without additional purification at an initial nearly neutral pH of 6.4 of MG and 6.6 for OOSS dye at room temperature and the TC supplied by Sigma-Aldrich and its aqueous solution pH is 7.2. The solutions pH is adjusted when studying the pH parameter to the desired values. A stock solution of a concentration of 1000 ppm was prepared and then diluted to the desired concentrations as needed. 100 mL of dye-containing solution was added into a glass container; then, the Fenton reagent at specific concentrations was added after pH adjustment in order to demonstrate the effect of the heterogeneous Fenton reaction. The as-synthesized Co/Fe@C was then added to the mixture as the catalyst source of the Fenton reaction. Subsequently, hydrogen peroxide (30% w/v) was poured into the solution to initiate the Fenton’s reagent reaction for oxidizing the wastewater. Notably, the pH value of the synthetic wastewater solution was adjusted to the desired values, if needed, by using diluted sulfuric acid or sodium hydroxide. All chemicals were supplied by Sigma-Aldrich of analytical grade and used as received without further treatment.

Subsequently, the solution undergoes of magnetic stirring to assure mixing and dispersion in the absence of light at ambient temperature (≈ 25 ◦C) in order to achieve the oxidation. Degradation experiments were completed in dark conditions to avoid the possible photocatalysis effect due to lab stray lights.

Analysis is then carried out at varies time intervals the spectrophotometric analysis to attain the remaining residual pollutant in the solution. Prior analysis the solution was initially filtered with a micro filter (0.45 μm) to remove any remains catalyst. The concentration of pollutants (TC, OOSS and MG) was evaluated by UV–vis spectrophotometer, Unico UV-2100 model, USA, corresponding to the maximum wavelength of 400, 410 and 615 nm at specific time intervals, respectively. All parameters affecting the oxidation process were also assessed. The oxidation efficiency is calculated according to the following equation:

1 $$\:OXidation\:\:Efficiency\:\left(\varvec{\%}\right)=\left[\:\frac{{\varvec{C}}_{\varvec{o}}-{\varvec{C}}_{\varvec{t}}}{{\varvec{C}}_{\varvec{o}}}\right]$$.

where, C_o_ represents the initial concentration of the contaminant, while C_t_ denotes the contaminant concentration at certain time t, in mg/L. Also, the samples’ pH was adjusted, when needed; by using a digital pH meter Model AD1030, Adwa instrument, Hungary. The graphical illustration of the experimental diagram is displayed in Fig. 1.

Furthermore, the reusability assessment is performed to verify the structural stability of Co/Fe@C catalyst. In such test, the catalyst was collected after each cycle of treatment by magnet (a neodymium permanent grade N42 magnet (surface field ~ 0.4 T)) and thereby followed by successive distilled water washing prior oven drying at 105 °C before being utilized for the subsequent catalytic cycle.

## Results and discussions

### Structural and morphological characterizations

#### XRD

**Fig. 2:**
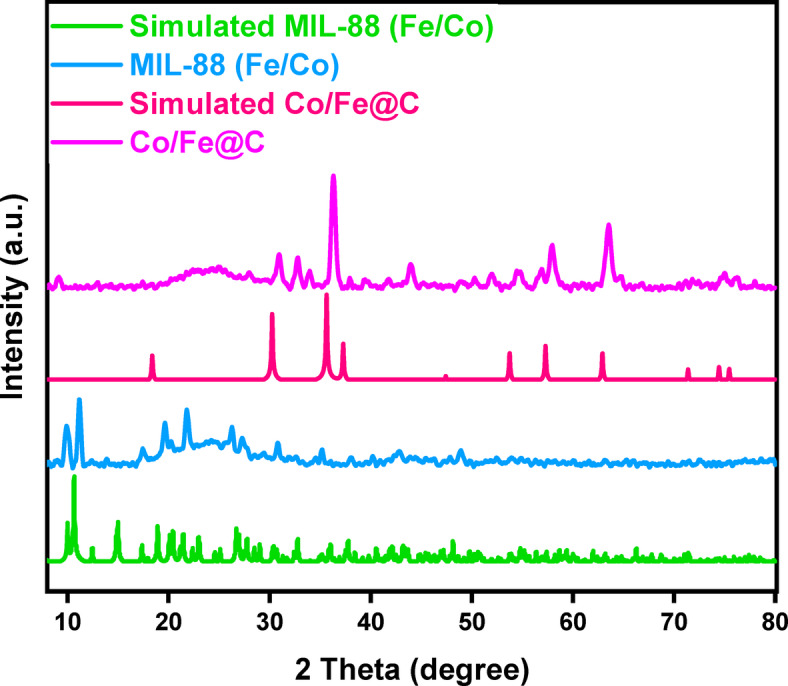
X-ray diffraction patterns of MIL-88B (Fe/Co) and Co/Fe@C

Figure 2 displays the MIL-88B (Fe/Co) XRD pattern. The observed 2θ values align with the simulation of single crystal data in the published literature^34^. This indicates that MIL-88B (Fe/Co) matrix allocates equal random Fe and Co. According to the previous work^34^, the breathing effect often significantly affects the XRD patterns of the phases by changing the cell volume and symmetry. In order to evaluate the crystallinity and phase purity of the prepared MIL-88B (Fe/Co), X-ray powder diffraction (XRD) was recorded for the MOF prior the carbonization process. Such investigation reveals the distinctive sharp peaks at 2θ of 9.99◦, 11.12◦, 17.52◦, 19.40◦, 21.90◦, and 27.05◦, which are in good agreement with those reported for simulated MIL-88B (Fe/Co) in earlier research^35^. Also, calcined MIL-88B (Fe/Co) catalyst revealed typical peaks of cobalt ferrite, most of that are not observable in the pristine catalyst due to the pyrolysis at high temperature. Such data is in consistent with the results of Fenton-like reactions described in the research. The diffraction pattern of the Co Fe alloy phase (JCPDS No. 49-1568) for the Co/Fe@C sample shows five distinct diffraction peaks at 30.6◦, 36.1◦, 44.08◦, 56.93◦, and 63.48◦^36^.

Furthermore, the crystallite size (D) of the MOF material prior and after carbonization are evaluated via Scherrer’s equation ($\:\:\text{D}=0.94{\uplambda\:}/{\upbeta\:}\text{c}\text{o}\text{s}{\uptheta\:}$) from the XRD dominating peak where β is the full width at half maximum intensity of the peak and θ is the diffraction angle (Scherrer (1918). The results showed that the crystallite size of the materials are 31.8 nm and 16.7 nm for MIL-88 (Fe/Co) and Co/Fe@C, respectively^37,38^.

#### SEM

**Fig. 3:**
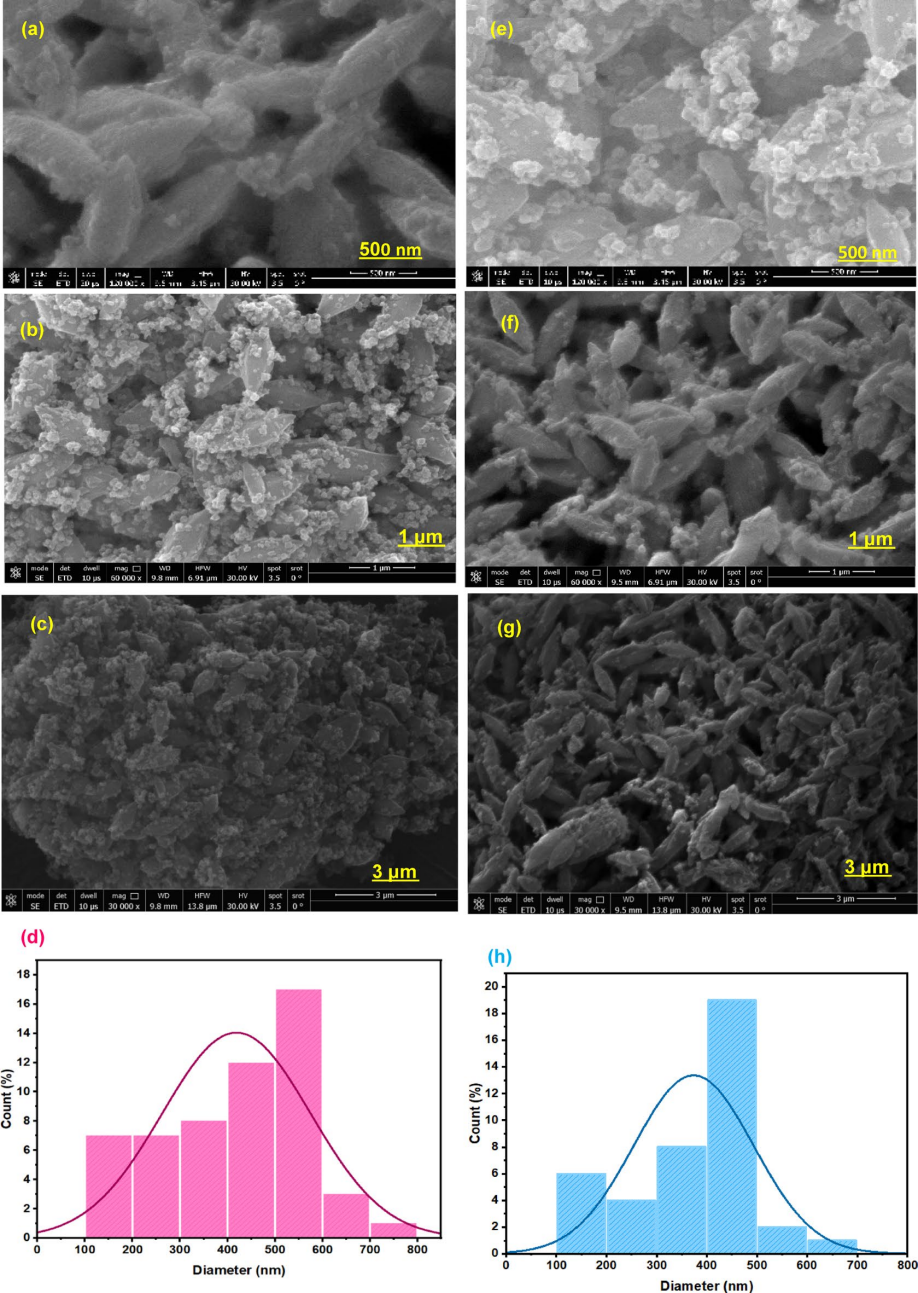
SEM images of (**a**, **b** and **c**) pristine MIL-88B and (**e**, **f** and **g**) Co/Fe@C at various magnifications, and their corresponding histogram-based particle size distribution (**d** and **h**, respectively)

SEM morphology of the Fe-based MOF (MIL-88B) as a model MOF precursor is attained. Figures (3a-c) shows the typical SEM morphology of spindle-like MIL-88B with a uniform size of 106–746 nm range (Fig. 3d and h). This signifies a reasonable size to offer high surface area Co/Fe@C composite material particles to be an efficient Fenton catalyst for the pollutant elimination through oxidation. According to the MIL-88B powder X-ray diffraction (PXRD) pattern (Fig. 2), materials with high crystallinity form in a homogeneous phase, which makes them especially efficient for water treatment^39^. Figure 3 (e-g) exhibited SEM images with varied scales of MIL-88B after carbonization at 500◦C labelled as Co/Fe@C-500.

The Co/Fe@C-500 shows a spindle-like nanocrystals similar to the pristine MOF with the particle size range about 132–695 nm (Fig. 3d) with no significant change in the morphology. Comparing the SEM images after and prior carbonization the surface texture shows more pores in their texture. This could be associated with regulated thermal treatment maintains porosity as well as promoting the formation of conductive carbon matrices embedded with scattered metal species. Such treatment is superior and beneficial in the catalytic oxidation applications since it provides both active site accessibility and improved electron transfer pathways^40^.

Further, the average particle size and is calculated and revealed that 419 nm with a standard deviation of 156.2 is the average particle size of MOF (MIL-88 (Fe/Co)) material, whereas, the Co/Fe@C composite signifies a 374 nm with 119.4 standard deviation. This confirms the increase in surface area after carbonization^41^.

#### VSM

**Fig. 4:**
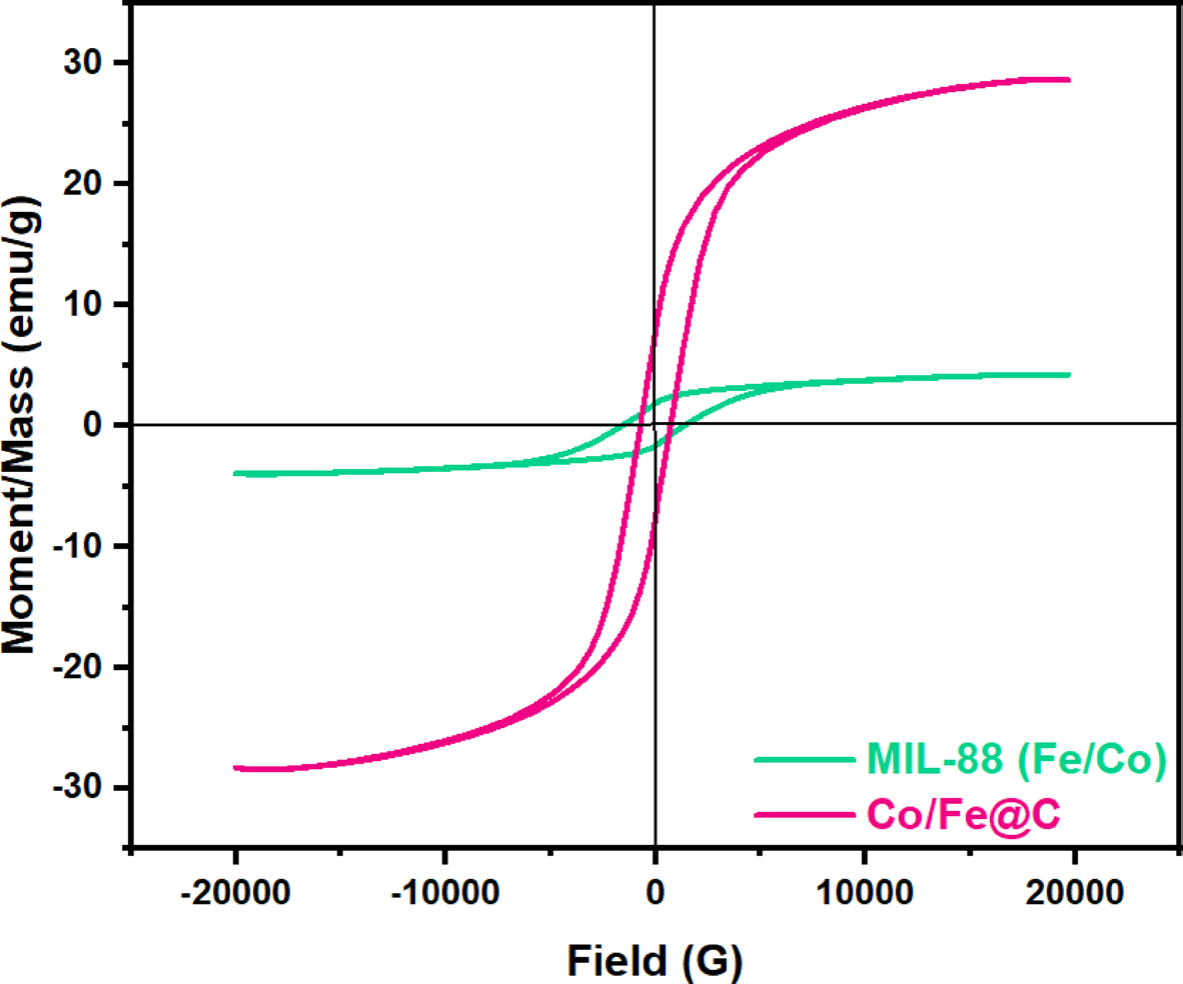
Magnetic hysteresis loop of the VSM studies and magnetic properties

The magnetic properties of Co/Fe@C and Fe/Co-MIL-88B are described by means of vibrating sample magnetometer (VSM) investigation. It is clear from the VSM plots (Fig. 4) that the saturation magnetization values of Co/Fe@C and Fe/Co-MIL-88B are 28.5 emu·g^−1^ and 4.1 emu·g^−1^, respectively according to eh data tabulated in Table 1. The coercivity and remanence values were determined to be zero from the magnetic hysteresis loop. In contrast to Fe/Co-MIL-88B, the results showed that Co/Fe@C exhibits a superparamagnetic nature.

A decrease in the crystallite size of the material according to the above-mentioned results for the material after carbonization results in an increase in saturation magnetization. The smaller particle size of Co/Fe@C MOF material compared with MIL-88 (Fe/Co) leads to a reduction in the inert layer causing saturation magnetization to increase due to the material carbonization. Also, the magnetic properties of the material depend upon the size of the MOF particles. As the deviation of reduction in the particle size is leading to the increase in the surface area. As the particle size decreases, the coercivity also declines that might convert the material to be a super paramagnetic MOF. It is also essential to mention that the increase in the magnetic properties of the material improves the MOF recyclability tendency that signifies the catalyst sustainability nature. This makes the introduced bimetallic MOFs exposed to magnetic fields and readily separated from the reaction mixture by magnetic attraction^42^ which facilitate the water treatment process. Paramagnetic property which allows easy separation of catalyst for possible reuse.

**Table 1:** Magnetization, retentivity and coercivity of MIL-88B and Co/Fe@C.

Sample code	Magnetization (emu/g)	Retentivity (emu/g)	Coercivity (G)
**MIL-88B** **Co/Fe@C**	4.11 28.579	1.75 7.7	1477 718.7

#### FTIR

**Fig. 5:**
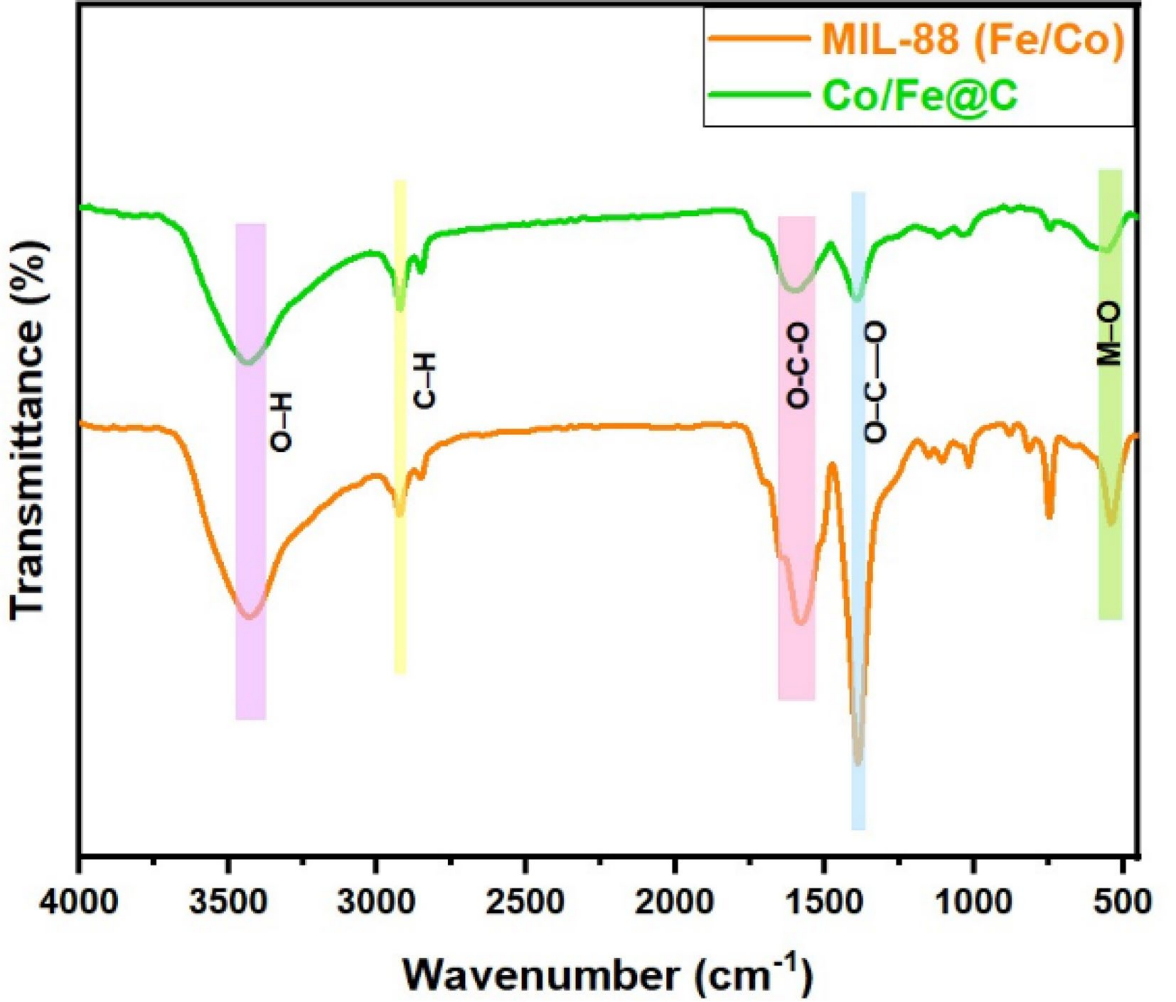
FTIR spectrum of MIL-88 (Fe/Co) and Co/Fe@C, wavenumbers range from 500 to 4000 cm^−1^

FTIR transmittance spectrum analysis is applied for the object of identifying the prepared MIL-88 (Fe/Co) and Co/Fe@C materials. The FTIR spectra of both MOF materials prior and after carbonization namely, MIL-88 (Fe/Co) and Co/Fe@C, respectively are displayed in Fig. 5. The FTIR data represents intensive peaks of MIL-88 (Fe/Co) observed at 540.75 cm^−1^ due to the M–O stretching that might be associated with the interplay between the carboxyl segment of terephthalic acid and pointing the Fe/Co atoms^26^. Two distinctive peaks are displayed at 1580 cm^−1^ and 1389 cm^−1^ that are attributed to the symmetric and antisymmetric O–C––O stretching frequency vibrations, respectively. Furthermore, extra distinct peaks at 2929 and 3430 cm^−1^ that attributing the existence of C–H bonding and O–H stretching mode that signifying the presence of benzene ring and/or adsorbed water species, respectively. Furthermore, the spectrum of the Co/Fe@C material represents the lost some peaks sharpness after carbonization. In addition, the carbonized MOF material signifying the increased the spectrum bands that appears at low wavenumber range of 600–500 cm^−1^. Such bands are corresponding to metal–oxygen vibrations of the novel created spinel-type Co/Fe@C^43^.

#### XPS

**Fig. 6:**
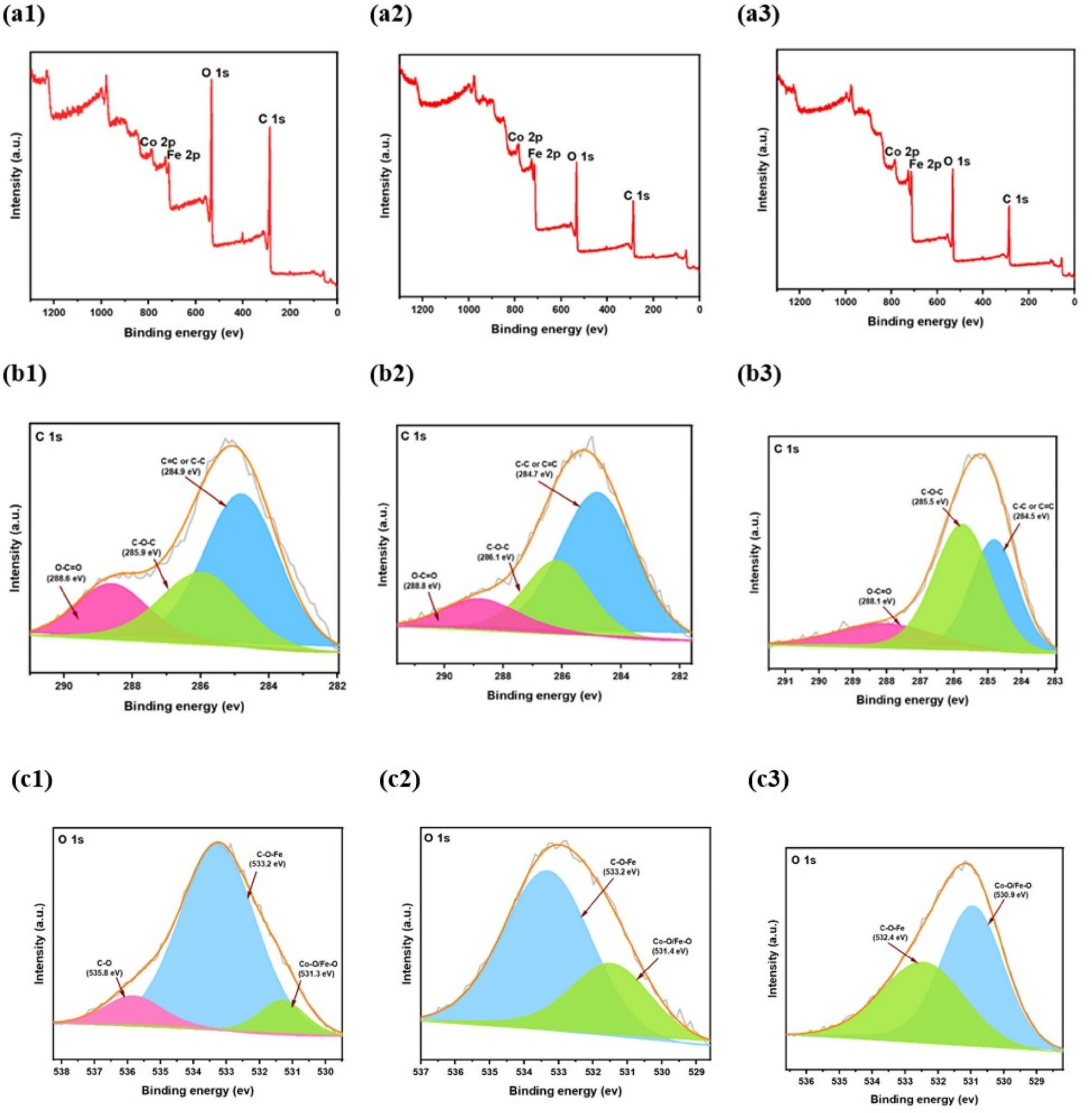

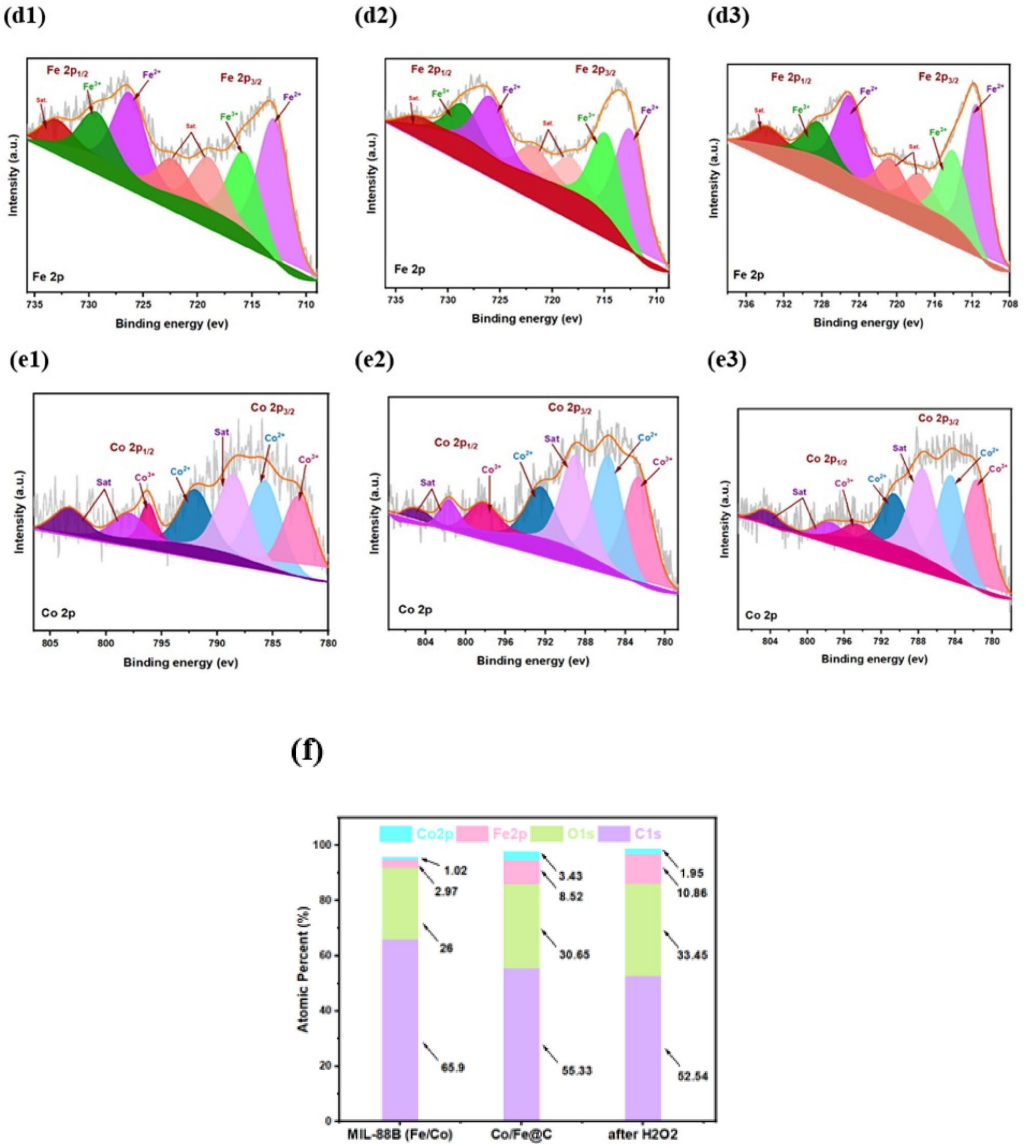
High-resolution scanning XPS spectra detailed line scans of the pristine MIL-88B (left column), Co/Fe@C (middle column) and the post-catalysis form (after catalysis with H₂O₂): (**a**) survey spectra of elements; (**b**) C 1s; (**c**) O 1s; (**d**) Fe 2p; (**e**) Co 2p; (**f**) element contents

The XPS analysis of the pristine, carbonized, and post-catalytic forms of MIL-88B (Fe, Co) provides a comprehensive understanding of the chemical changes that occur in such materials and the data displayed in Fig. 6. In the pristine material, XPS spectra of C 1 s, O 1 s, Fe 2p, and Co 2p regions indicate the presence of organic linkers, metal-oxygen interactions, and the initial oxidation states of iron and cobalt (Fig. 6(a). Du to carbonization, significant changes occur, including the formation of metal oxides and the transformation of the material’s structure. The catalytic process further alters the chemical environment, particularly in the metal centers, where cobalt and iron undergo redox changes that influence catalytic performance. In the following sections, we will discuss the changes observed in carbon, oxygen, iron, and cobalt individually and then correlate them to understand the overall transformation and catalytic behaviour of the MOF.

In pristine MIL-88B (Fe, Co), the XPS C 1 s spectrum (Fig. 6(b1) highlights the presence of sp² hybridized carbon (284.97 eV), sp³ carbon (285.95 eV), and carbonyl functional groups (288.61 eV), characteristic of the organic linkers stabilizing the MOF framework^43^. Upon carbonization, these peaks shift to 284.78 eV, 286.14 eV, and 288.83 eV, indicating the formation of oxygenated carbon species, such as C–O and C = O. This transformation is critical for creating a conductive carbon matrix that enhances electron transfer and stabilizes catalytic sites as illustrated in (Fig. 6(b2). Post-catalysis, the peaks at 284.56 eV, 285.53 eV, and 288.11 eV suggest further oxidation of carbon, likely due to the generation of reactive oxygen species (ROS) during catalytic decomposition of H_2_O_2_ as indicated in (Fig. 6(b3). These findings underscore the dual role of carbon as a structural element and an active participant in the catalytic mechanism.

The O 1 s spectrum reveals significant chemical evolution across the transformation stages. In the pristine state (Fig. 6(c1), peaks at 533.25 eV, 535.83 eV, and 531.3 eV correspond to lattice oxygen, adsorbed water/hydroxyl groups, and metal-bound oxygen (Fe–O, Co–O), respectively^44^. Carbonization shifts these peaks to 533.29 eV and 531.45 eV, reflecting the formation of active metal oxide species, such as CoO and Fe_2_O_3_, which are instrumental in H₂O₂ activation (Fig. 6(c2). Post-catalysis, the peaks at 530.93 eV and 532.43 eV indicate the formation of hydroxyl and peroxo intermediates, respectively (Fig. 6(c3). These intermediates play a critical role in ROS generation, emphasizing oxygen’s dual function as both a structural component and an active participant in catalysis.

Iron primarily stabilizes the MIL-88B (Fe, Co) structure through its redox equilibrium. The Fe 2p3/2 spectrum in the pristine state exhibits peaks at 712.89 eV and 726.23 eV, characteristic of Fe³⁺ species within the MOF framework as displayed in (Fig. 6(d1)^45^. In (Fig. 6(d2), carbonization results in shifts to 712.44 eV and 728.57 eV, indicating the formation of iron oxide species (Fe₂O₃) that indirectly enhance catalytic activity by supporting cobalt’s redox transitions^46^. Post-catalysis (Fig. 6(d3), the peaks at 711.47 eV and 724.98 eV suggest partial reduction of Fe^3+^ to Fe^2+^, which stabilizes the catalytic medium. Such analysis confirms the iron roe as a structural stabilizer essential for maintaining catalytic efficiency.

Additionally, cobalt serves as the primary catalytic site in MIL-88B (Fe, Co). In its pristine state (Fig. 6(e1), the Co 2p3/2 spectrum displays peaks at 785.69 eV and 782.65 eV, predominantly reflecting Co^2+^ species with minor contributions from Co^3+^. In (Fig. 6(e2), carbonization leads to shifts to 782.63 eV and 788.97 eV, indicative of mixed-valence cobalt oxides that exhibit enhanced redox activity^47^. Post-catalysis, the peaks at 781.6 eV and 790.58 eV suggest partial reduction of Co^3+^ to Co^2+^. Hence this maintaining the redox cycle and sustaining catalytic performance as depicted in (Fig. 6(e3). Figure 6(f) shows the clear drop in carbon content and increase in oxygen content of the calcined samples and post-catalytic form.

#### BET

**Fig. 7:**
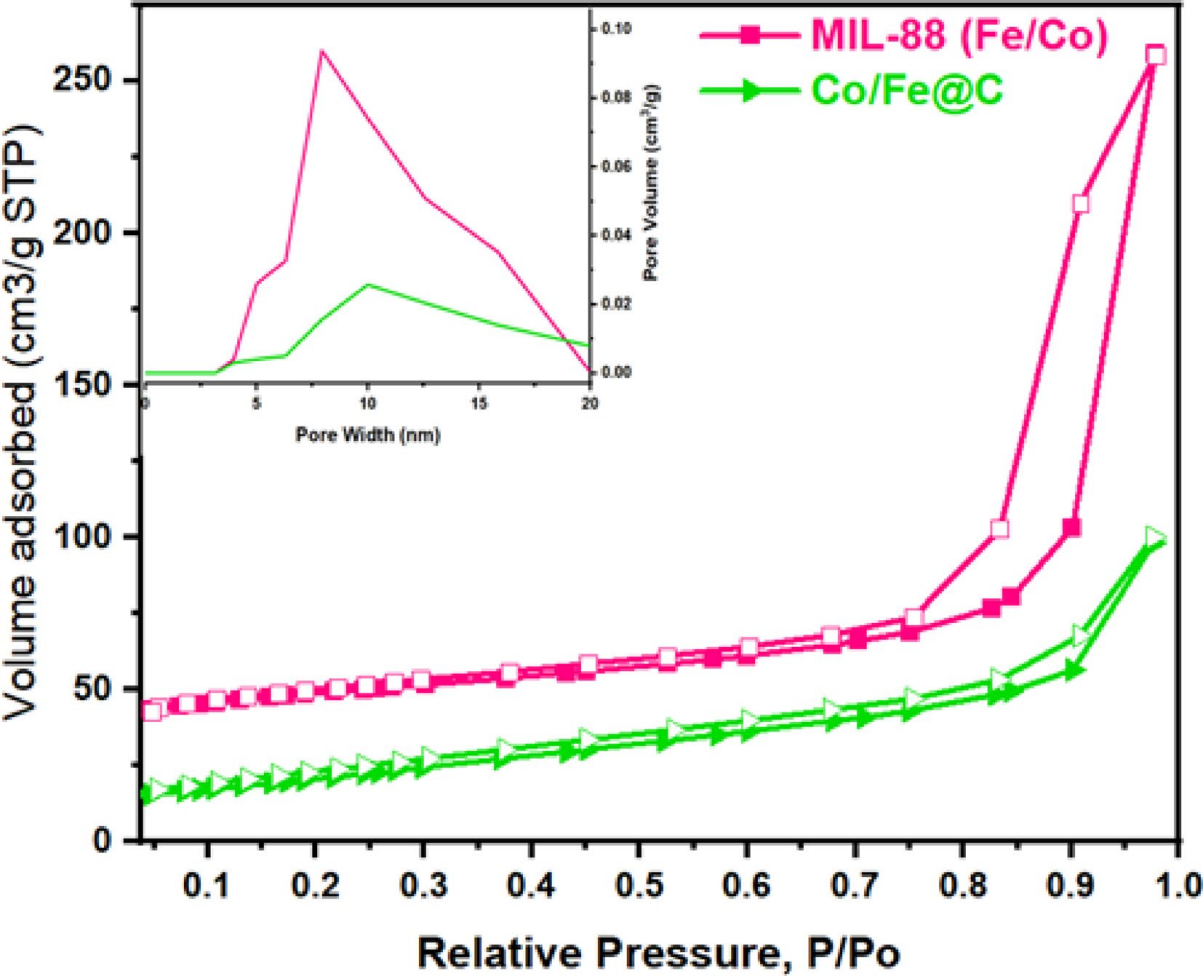
N_2_ adsorption–desorption isotherms for MIL-88 (Fe/Co) and Fe/Co@C (inset is pore size distribution curve)

In order to investigate the specific surface area and pore structures, nitrogen adsorption-desorption isotherms were investigated. Figure 7 illustrates that all sample isotherms exhibit a sharp increase at low relative pressures (< 0.01), followed by a slight enhancement of nitrogen uptake with increasing relative pressures and a hysteresis loop in the P/P0 range of 0.45 to 0.9, which is indicative of flexibility^48^.This relates to type-IV isotherms, which are characterized by the coexistence of mesopores and micropores^49^. Remarkably, BET study (displayed in Table 2) revealed that MIL-88 (Fe/Co) and Fe/Co@C had a nanopore crystalline structure, as shown by N_2_ sorption isotherms in Fig. 7. The BET specific surface areas of MIL-88 (Fe/Co) decrease as a result of MOF carbonization, attributed to the collapse of the porous framework. The MIL-88 (Fe/Co) was found to have an average pore size of 8.8 nm and a Brunauer–Emmett–Teller (BET) surface area value of 59 m^2^g^−1^. Following pyrolysis, Fe/Co@C exhibits the 39.5 m^2^g^−1^ surface area with an average size of 2.84 nm that achieve graphitization. By adjusting the carbonization of Fe/Co at 500 °C, Fe/Co@C exhibits a surface area that is comparable to MIL-88 (Fe/Co) with a slightly smaller mesopore size. Figure 7 pore size distribution (PSD) indicates that the materials are nanoporous and contain a significant number of mesopores. Thermal shock treatment of MIL-88 (Fe/Co) can optimize the hierarchical porous structure from the breakdown of organic ligands by adding Fe/Co metals. The structure of Fe/Co@C is approved to achieve the high dark degrading efficiency by such a developed porous structure. 

**Table 2:** BET surface area, average pore diameters, and pore volume of the MIL-88 (Fe/Co) MOF before and after carbonization.

Sample	BET area (m²/g)	Pore size (nm)	Pore volume (cm^3^/g)
MIL-88 (Fe/Co)	59.12	8.34	0.317
Co/Fe@C	39.54	2.84	0.123

### Fenton-Like catalytic oxidation of organic pollutants

#### Effect of oxidation time

Initially, to investigate all of the experimental variables affecting the oxidation reaction, the oxidation reaction time is assessed. In such experiment, the effect of various pollutants (MG, OOSS, and TC) mineralization is estimated in terms of their removal efficacy by the use of Co/Fe@C/H_2_O_2_ system and the solo Co/Fe@C system. To assess the optimal oxidation time and its impact on the system, experiments are carried out evaluating the reaction times ranged from 5 min to less than one hour, whereas all other parameters are kept constant (40 mg/L of catalyst, 400 mg/L H_2_O_2_ and pH of the natural solution). Further, all the experiments are undertaken at dark condition.

**Fig. 8:**
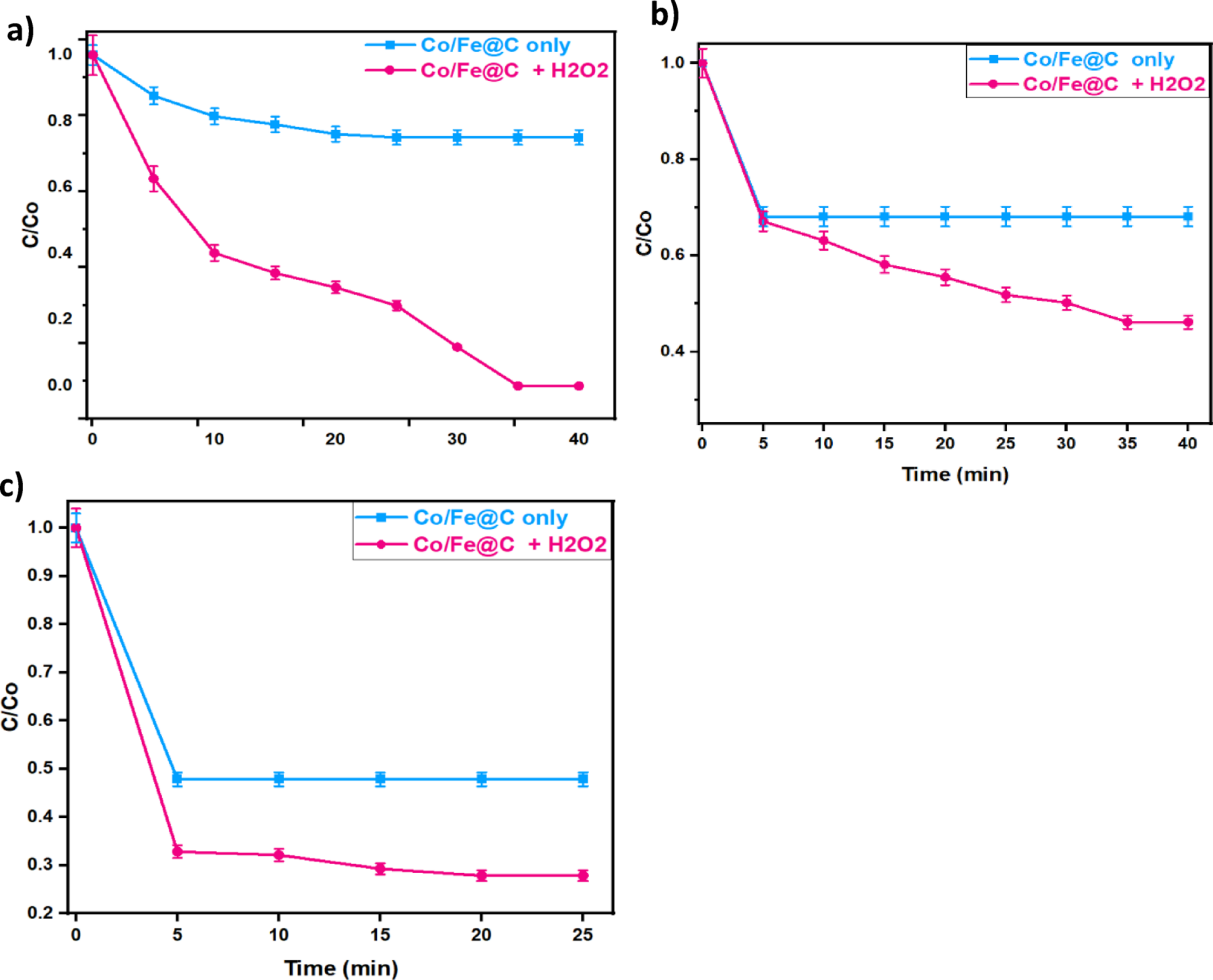
Effect of oxidation time on (**a**) MG (400 mg/L H_2_O_2_; 40 mg/L Co/Fe@C and pH 6.4), (**b**) OOSS (400 mg/L H_2_O_2_; 40 mg/L Co/Fe@C and pH 6.6) and (**c**) TC (400 mg/L H_2_O_2_; 40 mg/L Co/Fe@C and H 7.2)

The data displayed in Fig. 8. revealed that after 35 min, the cumulative removal rates reached to 25 and 32% for MG and OOSS dyes, as seen in Fig. 8a and b, respectively. It is noteworthy to mention that the removal rate is the highest at the initial reaction time, and subsequently steadily decreased rate is achieved. Also, the oxidation time profile of TC demonstrates 72% removals after 20 min of contact time as displayed in Fig. 8c. Notably, for all the studied systems superior pollutants removals are achieved when dual Co/Fe@C/H_2_O_2_ compared to the pristine Co/Fe@C system. such investigation verifies the role of the Fenton-like oxidation reaction^50^.

#### Effect of pollutant loading

To reach to the practical application life, it is crucial to investigate the influence of the pollutants load in the efficiency of the system to simulate industrial life. Accordingly, Fenton’s reagents were added to oxidize solutions of different concentrations of organic dyes and pharmaceutical pollutants under the following reaction conditions: (400 mg/L of H_2_O_2_, 40 mg/L of catalyst and pH 6.4) for MG solution, (400 mg/L H_2_O_2_, 40 mg/L of catalyst and pH 7.2) for OOSS dye and (400 mg/L H_2_O_2_, 10 mg/L of catalyst and pH 6.6) for TC.

**Fig. 9:**
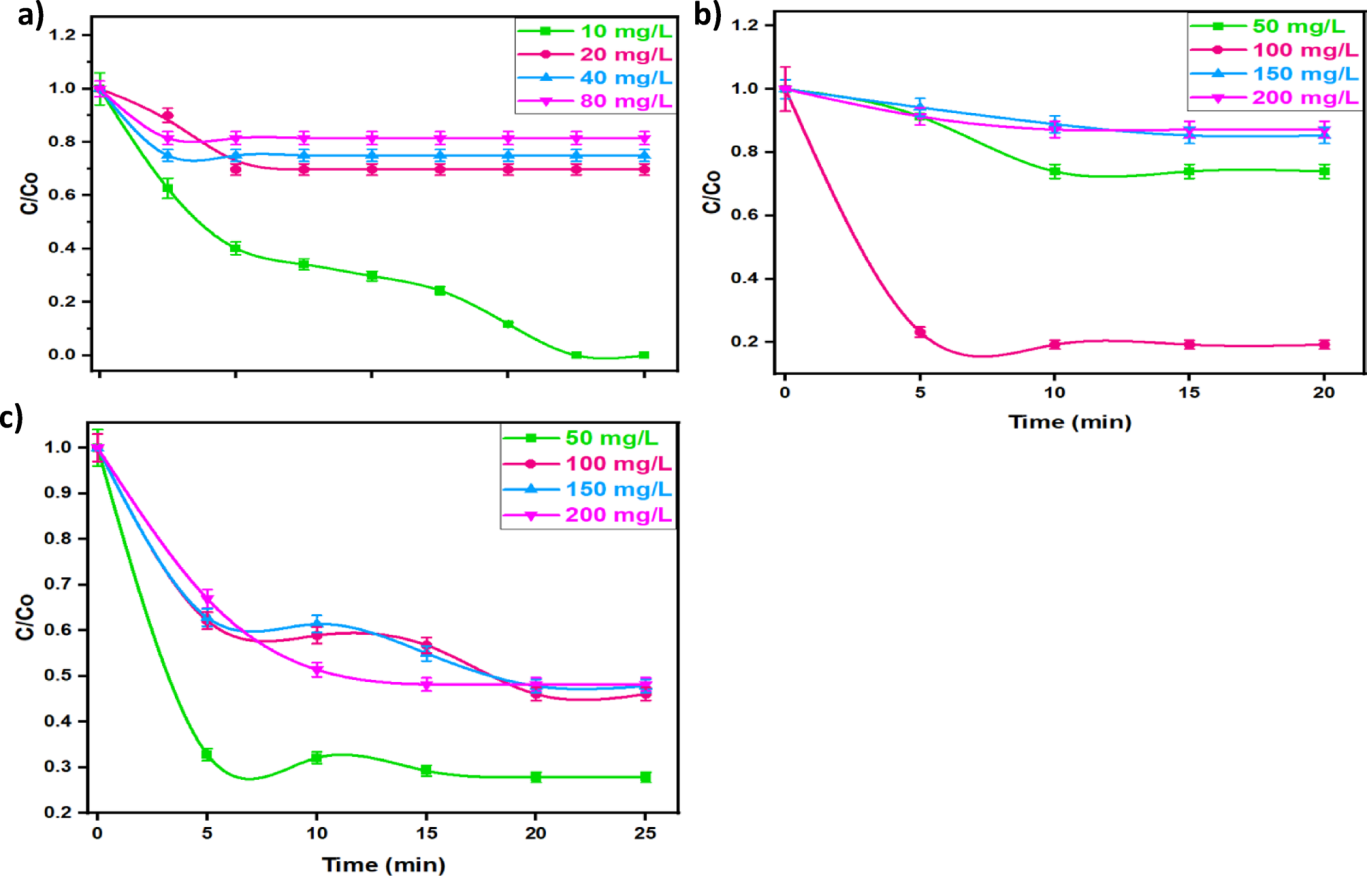
Effect of initial pollutant concentrations using modified Fenton’s system for (**a**) MG (H_2_O_2_ 400 mg/L; Co/Fe@C 40 mg/L; pH 6.4), (**b**) OOSS (H_2_O_2_ 400 mg/L; Co/Fe@C 10 mg/L; pH 3.0) and (**c**) TC (H_2_O_2_ 400 mg/L; Co/Fe@C 40 mg/L; pH 7.2)

The results displayed in Fig. 9 (a, b and c) revealed the effect of various concentrations on the oxidation system. The effect of MG dye concentration is ranged studied in the range of 10 to 80 mg/L, while OOSS and TC are in the range of 50 to 200 mg/L. according to the results displayed in Fig. 9, with increasing the initial concentrations, the oxidation rate for all pollutants is declined. With the time proceeding, MG dye removal rate could be reached to complete removal of 100% dye removal within After 35 min through the introduced dark-Fenton reaction for the low initial dye concentration reached to only 10 ppm. Whereas, when the dye concentration increases to 20, 40 and 80 ppm, the oxidation rate decreases to 30, 25 and 19%, respectively as displayed in Fig. 9a. Also, similar trend is achieved for OOSS dye. The oxidation rate is declined from 83% to only 12% when the dye concentration is increased from 50 to 200 ppm, respectively (Fig. 9b). Also, TC oxidation is achieved within 20 min of oxidation time that reached to 72% that corresponding to the 50-ppm concentration; however, when the concentration of TC increased (Fig. 9c), the oxidation efficiency is declined to 52% at the 200-ppm concentration. Such phenomenon is associated with the concentration of hydroxyl radicals, which remains constant, while the pollutant load is increased. Thus, the oxidation rate is deduced at the high concentrations of pollutants since the number of reagents are not sufficient for producing enough OH radicals. Previous work cited in literature recorded a similar trend in oxidizing various pollutants using Fenton based system^5,52,52^.

Thus, although a reduction in surface area of Fe/Co@C after carbonization is achieved according to the abovementioned data, it does not hinder its catalytic activity due to the change in pore structure. Furthermore, the presence of carbon in the material (Fe/Co@C) after carbonization also improves the catalytic activity of such catalyst compared to MIL-88 (Fe/Co). It is essential to mention that the reduction in surface area during the carbonization of MIL-88B (Fe/Co) does not hinder catalytic activity for pollutant oxidation. This is due to the well-dispersed Fe/Co@C within a synergistic carbon matrix creates a more effective catalytic environment. The key feature is the quality and accessibility of the active sites, their intrinsic reactivity, and the beneficial interactions with the carbon support, which collectively outweigh the effects in the overall surface area essential for the pollutant removals.

#### Modified Fenton-like’s multiple parameters, co/fe@c, H_2_O_2_ and pH effect

Hydrogen peroxide reagent decomposition in the presence of Co/Fe@C for generating ·OH radicals is essential for oxidizing the organic pollutants. But it is critical to keep both reagents H_2_O_2_ and Co/Fe@C concentrations minimal since the higher concentrations declines the oxidation efficiency. Consequently, to investigate the influence of Co/Fe@C catalyst on the modified Fenton’s oxidation of pollutants, experiments were undertaken to establish their influence on reaction kinetics.

##### Effect of catalyst dose

**Fig. 10:**
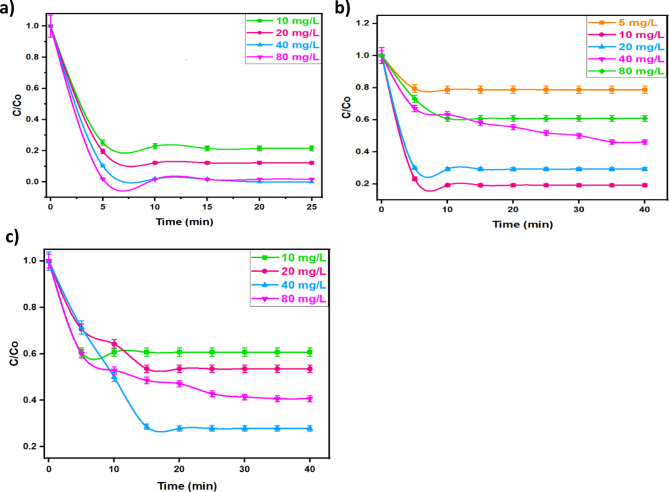
Effect of Co/Fe@C catalyst concentration on (**a**) MG (Dye_,_ 10 mg/L; H_2_O_2_ 400 mg/L; pH 6.4), (**b**) OOSS (Dye_,_ 100 mg/L; H_2_O_2_ 400 mg/L; pH 3.0) and (**c**) TC (Drug_,_ 50 mg/L; H_2_O_2_ 400 mg/L; pH 7.2)

To assess the influence of catalyst dose on the various pollutants removal rate, the Co/Fe@C concentration was increased in the reaction medium from 10 to 80 mg/L, whereas all other parameters are kept constant (400 mg/L for H_2_O_2_ concentration) and the initial pH is kept at 3.0 for OOSS and natural values for MG and TC. According to the data displayed in Fig. 10, for all the studied pollutants, increasing the Co/Fe@C concentration results in a high oxidation rate and the maximal reduction efficiency reached to 100, 72, 83% for MG dye, TC and OOSS, respectively, when the optimal concentration is added. However, extra Co/Fe@C concentration addition more than 40 mg/L when oxidizing MG and TC and more than 10 mg/L when oxidizing OOSS dye. This could be attributed by the ˙OH radicals are trapped by excess Co/Fe@C concentration. Thus, extra addition of the Co/Fe@C concentration speciation in the aqueous medium hinders the OH radicals’ performance. Such optimal dose of the Co/Fe@C catalyst is linked to maximal increase in the number of active sites, which encourages the synthesis of •OH during the Fenton-Like process^53^. Also, the presence of catalyst in excess results an agglomeration that hinders its activity since the available surface area for producing OH radicals is declined^54^. Hence, the extra Co/Fe@C presence is acting as OH radical’s scavenger rather than a producer.

##### Effect of H2O2 concentration

**Fig. 11:**
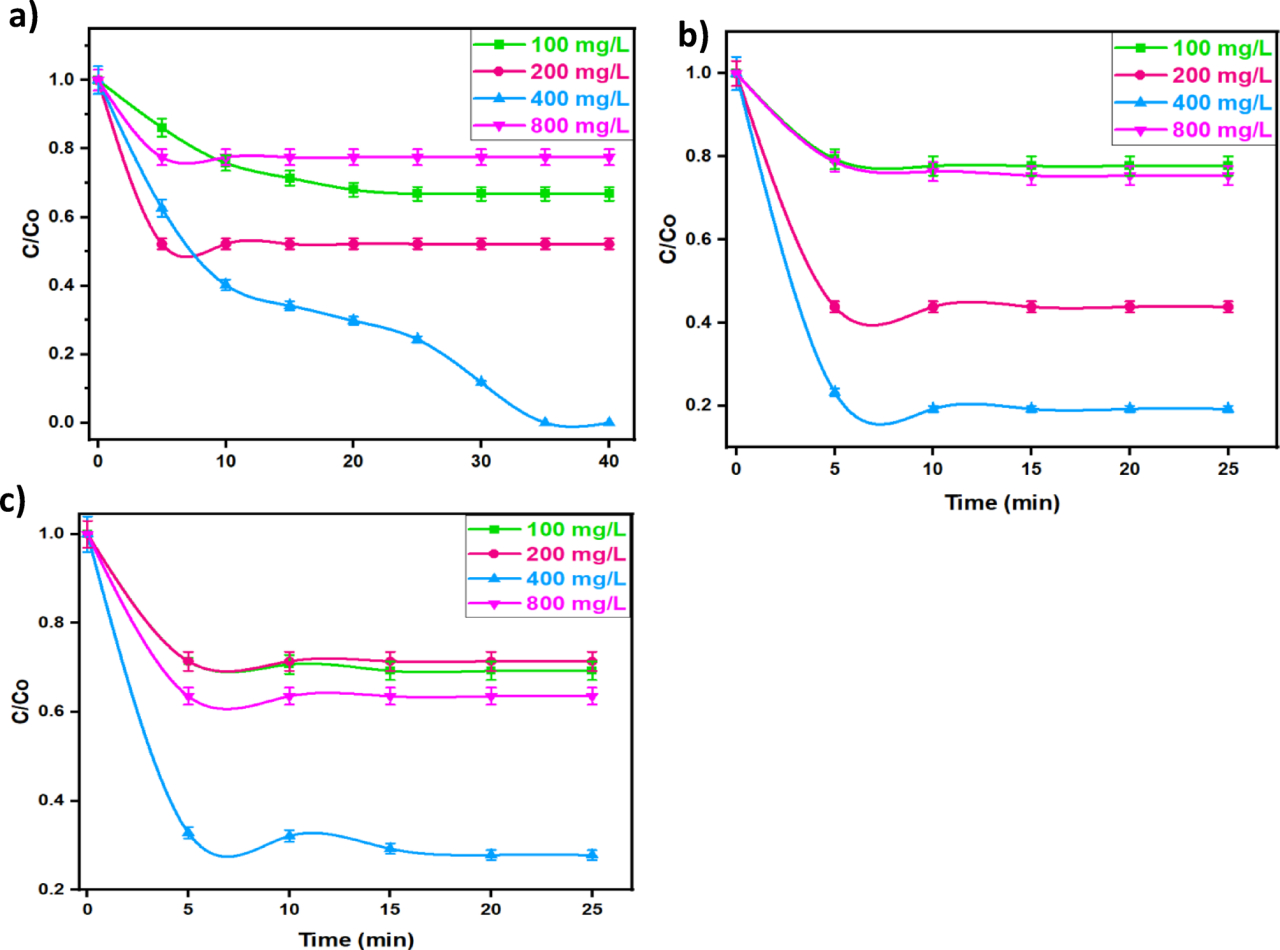
Effect of different hydrogen peroxide doses on (**a**) MG (Dye_,_ 10 mg/L; catalyst 40 mg/L; pH 3.0), (**b**) OOSS (Dye_,_ 100 mg/L; catalyst 10 mg/L; pH 3.0) and (**c**) TC (Drug_,_ 50 mg/L; catalyst 40 mg/L; pH 7.2)

The presence of hydrogen peroxide in the Fenton system is crucial. In this regard, varying hydrogen peroxide concentration is investigated on the rate of oxidation of the studied pollutants, namely TC drug, MG dye, and OOSS dye. By keeping all the other conditions constant, H_2_O_2_ dosages are varied from 100 to 800 mg/L. According to the data displayed in Fig. 11 (a, b, and c), increasing the hydrogen peroxide concentration for all pollutants results in an increase in the oxidation efficiency until the 400 mg/L addition of such reagent. Whereas, further increase in hydrogen peroxide more than such 400 mg/L dose, the result in a deduction in the oxidation rate. This could be attributed by excess OH radicals are generated in the reaction medium when the optimal dose of H_2_O_2_ is expected to achieve a high rate of oxidation. This confirms that H_2_O_2_ might be in an optimal existence. However, the existence of hydrogen peroxide more than the optimal occurrence results in the formation of perhydroxyl radical (HO_2_˙), which is less reactive than OH radicals. Hence, the oxidation efficacy is reduced for all pollutants^55^.

##### Effect of initial pH

**Fig.12:**
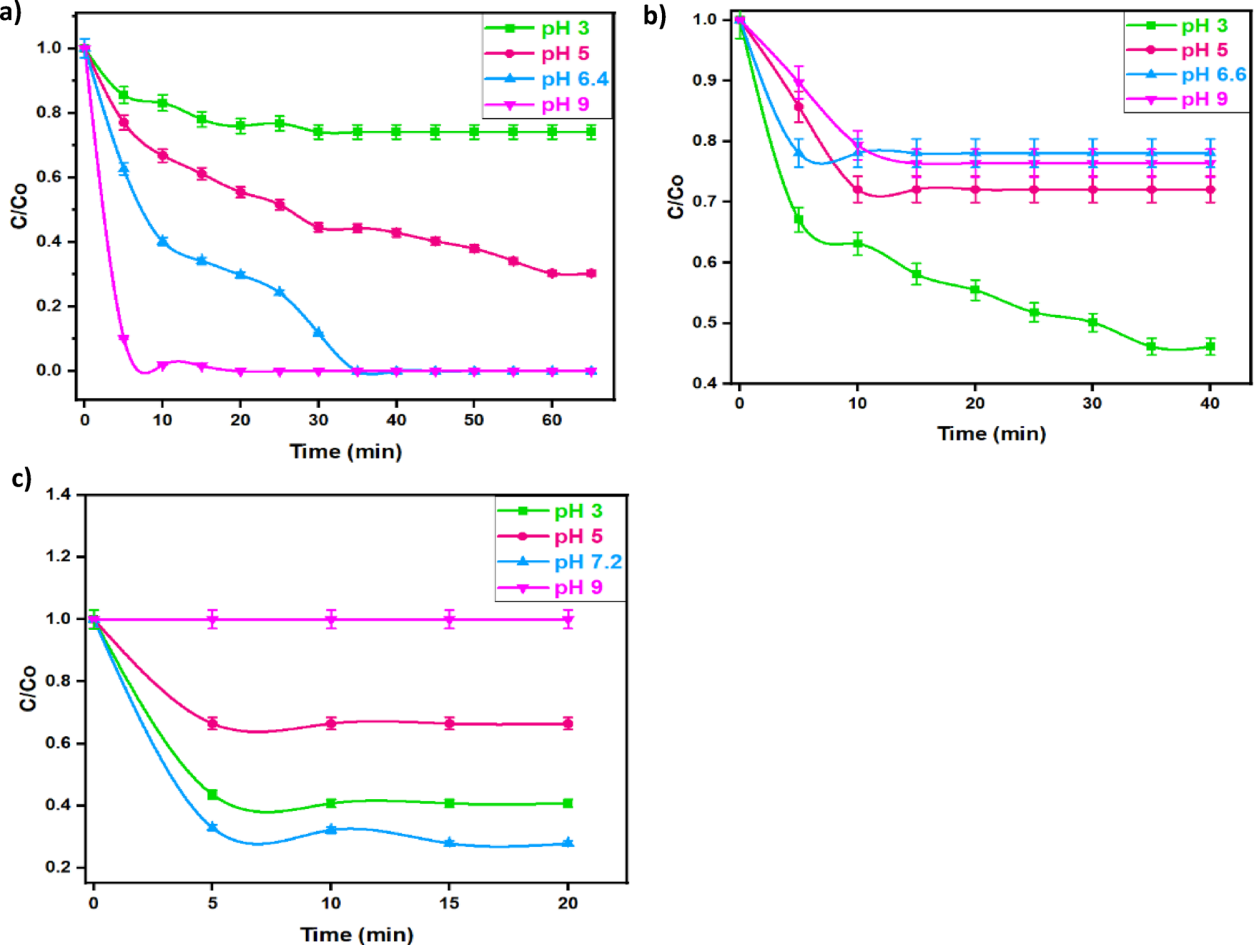
Effect of different solution pH at 400 mg/L of H_2_O_2_and catalyst on (**a**) MG (Dye_,_ 10 mg/L); (**b**) OOSS (Dye_,_ 100 mg/L) and (**c**) TC (Drug_,_ 50 mg/L) pollutants removal during oxidation

The pH effect in the Fenton reaction plays a crucial role that influences the rate of oxidation of pollutants molecules. Thus, various experiments are conducted to check pH influence by varying the initial pH of the aqueous effluent pH in the range of 3.0 to 9.0 to determine its impact on the oxidation of MG, OOSS, and TC using the introduced Fenton-Like reagent (Co/Fe@C/H_2_O_2_), as illustrated in Fig. 12 (a-c). Figure 12a shows that increasing the pH results in a high performance of Co/Fe@C/H_2_O_2_ and extra dye is oxidized and the optimal pH is recorded at 9.0. This could be attributed by the MOF surface become more negatively charged in the alkaline medium. Also, the alkaline pH helps in deprotonation the medium and partial dissolution of the MOF structures is attained that maximize its catalytic activity. Furthermore, stronger interaction between the more negatively charged MOFs and MG as a type of cationic dye is achieved^56^. Although, MG dye oxidation is superior at pH 9 (100% removal within 20 min), also natural pH of the dye solution (6.4) exhibited a complete dye removal reached at 35 min. Additionally, similar trend is achieved when TC is oxidized (Fig. 12c) at its natural pH (7.2) achieved 72% of TC removal. However, when OOSS is oxidized with such reagent, the acidic pH condition is favourable. This is due to rate of generating ·OH radicals is in excess since the rate of hydrogen peroxide decomposition is high while the ·OH radicals recombination reaction is low. Furthermore, the OOSS is negatively charged dye due to the presence of sulphate group, thereby the acidic pH conditions help in high dye dissociation in the solution and the result is a good electrostatic interaction between the negative dye molecules and positively charged MOF^52^. Additionally, it is noteworthy to mention that such investigation is confirming the principle of hard, soft acid-base concept explains that MOFs were constructed from hard acid (carboxylate group), and hard base (high valence metal ion, i.e. Co^3+^), which provides good stability near neutral or may be acidic pH conditions^57^. Previous reports in literature verifying such investigation^58^.

To promote a full understanding of the pH variation on the oxidation system, the point of zero charge (pH_PZC_) for Co/Fe@C is conducted. It assists in understanding the effect of oxidation process and describes the condition at which the surface electrical charge density of the catalyst is equal to zero. It is determined by taking solutions with initial pH in the range 2, 4, 6, 8, 10 and 12. The desired pH was obtained by adjusting the solution using 0.01 M HCl or NaOH^59^. The solutions were then continuously stirred for 24 h. The final pH values were recorded and compared with the initial pH values. Then, the pH value is measured and the change in pH (ΔpH) is equal to zero value is corresponding to the point of zero charge (pH_PZC_). But the catalyst surface becomes negatively charged when the recorded pH value is higher than the pH_PZC_ and vice versa, the lower pH value less than the pH_PZC_ is signifying the positively charged surface.

**Fig. 13:**
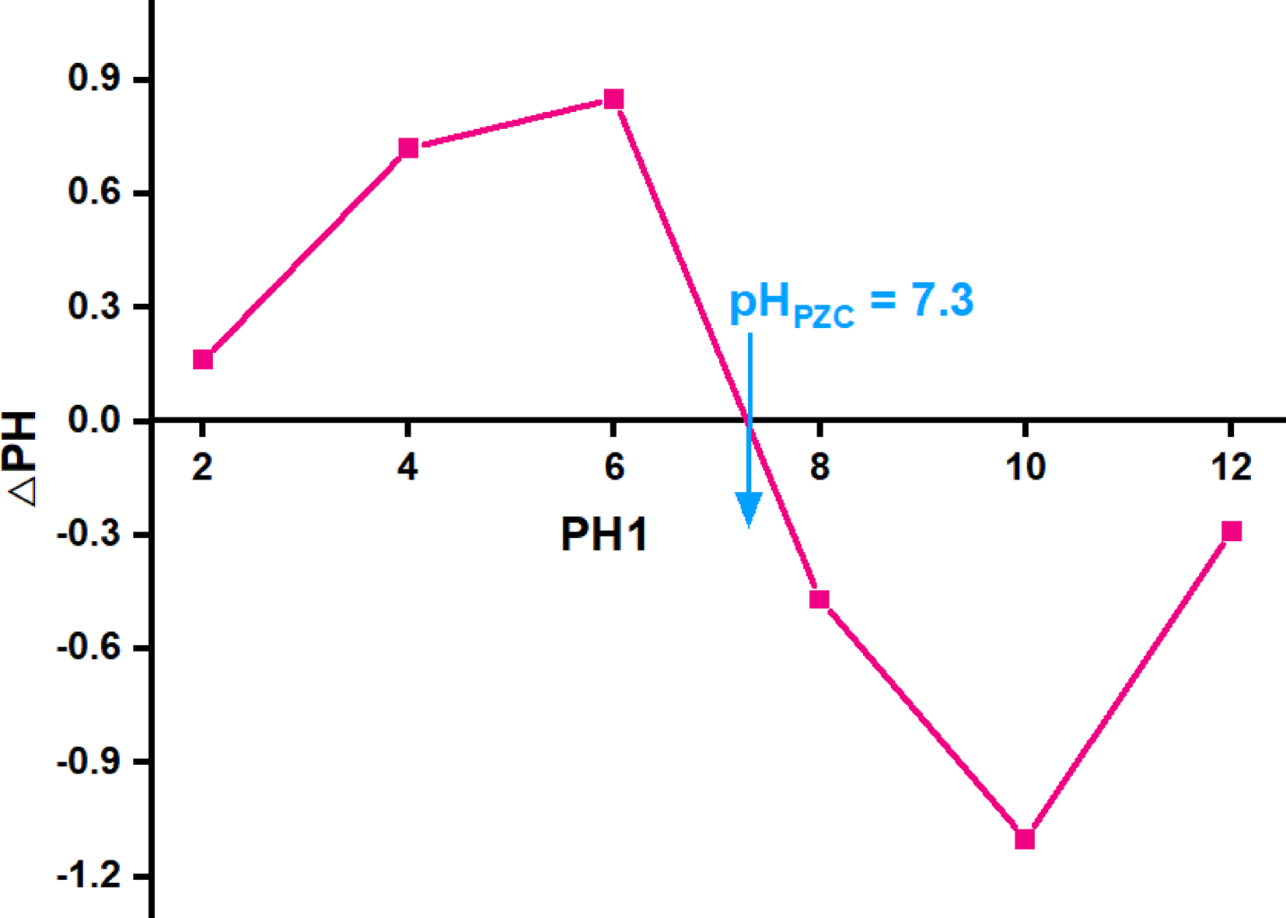
Point of Zero charge (pH_PZC_) of Co/Fe@C catalyst

In the present study, the results displayed in Fig. 13 exhibited that the pH_PZC_ for Co/Fe@C is displayed as 7.3. Thus, when the pH value is higher than the point of zero charge (pH_PZC_), the MOF surface attains a negative charge. However, at a pH lower than pH_PZC_, MOF surface possesses a positive charge. Notably, the catalyst surface is thereby negatively charged that verifies the higher MOF catalyst activity at pH near neutral. The oxidation efficiency reached to 100, 81 and 72%, MG, OOSS and TC, respectively. Since OOSS is an anionic dye, the electrostatic repulsion between OOSS and the negatively catalyst surface charge is thereby limiting the dye oxidation. Thus, a lower oxidation rate is achieved. However, opposite trend is achieved for MG dye oxidaiton. Positive holes are the primary oxidation species at pH < pH_PZC_, while hydroxyl radicals (OH−) are primarily responsible for the oxidation process when pH ≥ pH_PZC_^60^.

**Fig. 14:**
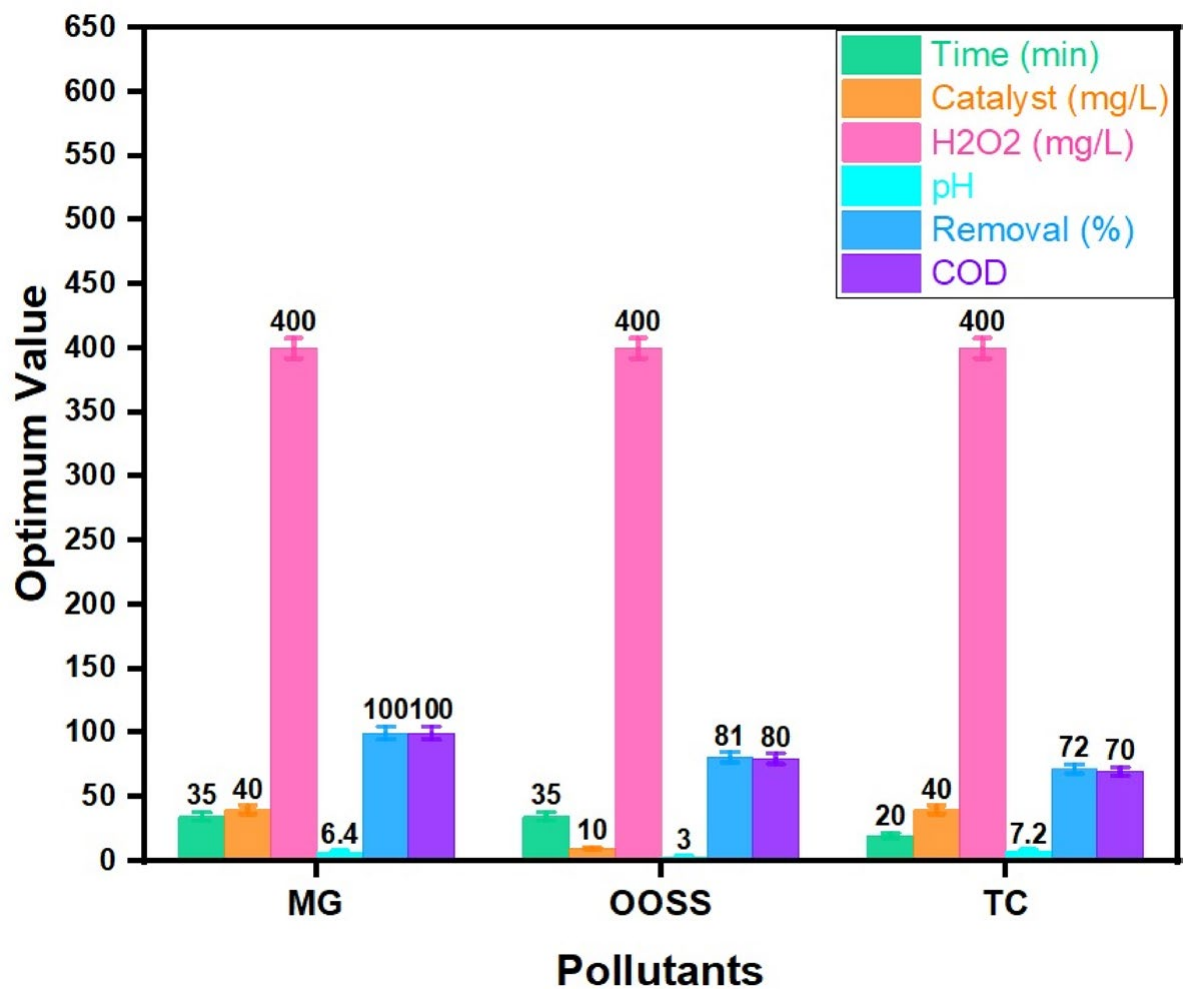
Comparsion of the optimum conditions for oxidizing diffent pollutants using Co/Fe@C based Fenton system

The optimum conditions for all pollutants are displayed for comparing the optimum conditions for each pollutant. According to the results in Fig. 14, the oxidation reaction time is ranged from 20 to 35 min according the pollutant degraded however, further oxidation time according to the above-mentioned data is not leading to a further increase in the oxidation process whereas, it is noteworthy to mention that 35 min of oxidation time could reach to 100% removal in the case of MG. Also, the optimum pH is ranged from the acidic to natural pH of the pollutant since it is associated to the OH radicals’ production and thereby affects the rate of oxidation efficiency. Furthermore, as displayed in Fig. 13, the point of zero charge is changed according to the pollutant type, thereby the acidic pH is the optimum when the OOSS is essential to oxidize whereas TC and MG their natural pH is sufficient for oxidation. Whilst, the optimum hydrogen peroxide is 400 mg/L for all pollutants this means the hydrogen peroxide is in excess in the reaction medium rather than the catalyst dose. This is due to the catalyst activation is enhanced when the peroxide is in excess. The MOF catalyst reacts in less activity when the hydrogen peroxide is not in the optimal dose. Such investigation is in the same trend of the previously cited reports in treating textile dying effluent discharge^61^.

Additionally, the catalyst dose is ranged from 10 to 40 mg/L according to the type of pollutant oxidized. In the case of the oxidation process of the OOSS dye the catalyst is 10 mg/L. But, the Fenton oxidation system for oxidizing TC and MG is using a higher dose of the catalyst of 40 mg/L due to the increase in the produced ^•^OH radicals by using higher concentrations of the oxidant. This means a 4-fold of the Co/Fe@C is applied for oxidizing TC (72% oxidation efficiency) and MG (100% oxidation efficiency) compared to OOSS (81% oxidation efficiency). This could be associated with the type of the pollutant oxidized. As evidenced by data from the previous reported literature^62^, increasing the dose of catalyst increases the efficiency of the catalytic decomposition of hydrogen peroxide, but their presence in the reaction medium in excess than the optimal concentration might be acts as a so-called radical scavenger, causing the ^•^OH generated to bond together and hinders the reaction rate^37,63^.

Furthermore, the Chemical Oxygen Demand (COD) prior and after oxidation is measured and the data displayed in Fig. 14 confirmed a complete MG removal in terms of COD oxidation and the COD reduced from 19 mg-COD/L to a complete COD reduction. However, only 80 and 70% removals are achieved for OOSS and TC pollutants, respectively. Thus, such data verifies the pollutant oxidation displayed in terms of concentration of pollutant.

#### Oxidation kinetics

From the theoretical and practical point of view, it is esesntial to assess large-scale treatment capacity. Studying the reaction kinetics is essential to full understanding the performance of oxidation reaction. As the industrial wastewater dischage might be disposed off at different concentrations, the effect of variation of concentration of each studied target pollutant, MG, OOSS and TC, with time through the Fenton-like based on Co/Fe@C oxidation reaction is analyzed in order to investigate the kinetics of the reaction. The general kinetic rate law for reaction of the pollutants is investigated according to the linearized equation form that are dispalyed in Table 3. Therefore, the oxidation of various pollutants using Co/Fe@C-Fenton oxidation process is investigated using zero-, first-, and second-order reaction kinetic models. Also, the kinetic rate constants and the reaction half-life (t_1/2_) for Co/Fe@C Fenton-Like system is also tabulated in Table 3.

Based on the assessment of the regression coefficients values (*R*^*2*^) exhibited in Table 3, it is seen that values of *R*^*2*^ for the first-order and second-order reaction rate were significant, the data is well fuitted for first- or second-order that most appropriate signifies the experimental data. Alhtough in some cases the first and second order fitting the data, the best model fit could be signified for the second-order reaction kinetics. The second-order regression coeffceint for all pollutants is ranged from 0.70 to 0.99. Also, it is naotably that the second-order model rate constants are increased with the catalyst dose increase from 10 to 80 mg/L. This could be attributed by the increase in the catalyst dose increase the reaction rate since the OH radicals’ production is increased. However, with the catalyst dose increase to 80 mg/L, a decline in tthe kinetic constants is achived again since the increase in the catalyst excess than the optimal concentration hinders the OH radicals production^25^.

As displayed in Table 3, the half-life time (*t*_*1/2*_) declines with increasing the catalyst dose and the least half-life time (*t*_*1/2*_) is corresponding to the optimal catalyst dose. This could be illustrated by increasing the MOF catalyst dose improves the oxidation reaction, however, further increase in the catalyst dose hinders the oxidation reaction since the catalyst is settled in the reaction medium rather than reacting with the hydrogen peroxide and generating the OH radicals. Such radicals are the main responsible of the reaction. Thus, the reaction is declined and the half-life time in then increases. Additionally, the lesser the catalyst dose less than the optimal value, the hydroxyl OH radicals output are not adequate to oxidize the varied pollutants at the too low concentration^17^. There is slightly difference of the *K*_*2*_ values for varied pollutant samples are related to the difference in the structure feature of the pollutants^64^.

**Table 3:** Kinetic parameters of various kinetic models for MG dye, OOSS dye and TC pharmaceutical pollutants’ oxidation via Co/Fe@C/H2O2.

Catalyst Conc. (mg L^−1^)	Zero-Order Kinetics			First-Order Kinetics			Second-Order Kinetics		
	k_0_ (mg L^−1^ min^−1^)	t_1/2_(min)	R^2^	k_1_(min^−1^)	t_1/2_(min)	R^2^	k_2_ (Lmg^−1^min^−1)^	t_1/2_(min)	R^2^
**MG dye**									
10	0.27	1.68	0.46	0.09	7.35	0.68	0.018	641.7	0.80
20	0.31	1.91	0.48	0.13	5.1	0.77	0.059	202.08	0.99
40	0.36	2.2	0.5	0.27	2.48	0.89	0.363	33.17	0.92
80	0.33	2.04	0.43	0.24	2.78	0.63	0.491	24.54	0.90
**OOSS dye**									
10	1.17	29.4	0.34	0.11	6.05	0.74	0.0017	58822	0.75
20	0.95	23.7	0.3	0.074	9.33	0.61	0.0007	14285	0.96
40	1.03	25.8	0.71	0.034	20.5	0.81	0.0003	333327	0.90
80	0.64	16.1	0.44	0.032	20.7	0.85	0.0002	499991	0.90
**TC pharmaceutical**									
**10**	0.26	6.5	0.3	0.02	34.65	0.50	0.0008	62475	0.70
**20**	0.43	10.7	0.57	0.03	22.72	0.89	0.0011	45436	0.77
**40**	0.77	19.3	0.65	0.06	9.98	0.95	0.0032	15618	0.90
**80**	0.53	13.4	0.61	0.034	20.2	0.78	0.0014	35700	0.90

$$*C_{t} = C_{o} - k_{0} t,\user2{ }**\user2{ }(C_{t} = C_{o} - e^{{k_{1} t}} ~,***\left( {\frac{1}{{C_{t} }}} \right) = \left( {\frac{1}{{C_{0} }}} \right) - k_{2} t,~$$ k0, k1, k2: kinetic rate constants of zero-, first- and second-reaction kinetic models; Co and Ct¬ : pollutant concentrations at initial and time t; t: time; R2: correlation coefficient; t1/2 half-life time. .

####  Comparison of data with literature

Comparison the contaminants removal using the modified Fenton systems, various composite iron catalyst sources for Fenton reaction from literature and compared with the current modified system from MOF based carbon is presented in Table 3. It can be concluded that the modified Fenton oxidation system based on the MOF based carbon from the current system showed an efficient treatment reached to 100% MG removal within only 30 min of reaction time. Although many other treatments showed high treatments efficiency (100%) from other treatment systems reported earlier have displayed their potential (Table 3) for various pollutants’ removal from wastewater, such systems possesses some disadvantage such as the cost of unsustainable chemical agents use makes the process costive besides the formation of toxic chemical products. Also, more reaction time is required for other process compared to only 30 min of reaction time through the current investigation. Moreover, the current investigation is based on the use of alum sludge waste augmented with magnetite nanoparticles, which make the composite recoverable and recyclable as a sustainable option for the modified Fenton source. Additionally, these groups of disadvantages are not associated with current oxidation technology recommended for pollutants oxidation. Hence, this current suggestive investigation is much better and cheaper especially when the recyclable MOF base carbon composite is used as the source of Fenton-like process. Additionally, the process is environment friendly compared to the other listed techniques in Table 3 that are not based on recyclable environmentally safe materials.

#### Recyclability

One of the most esteemed benefits of any heterogeneously Fenton based catalysed system is the catalyst reusability. But there is a lack in literature on the subject of carbonized MOF catalyst reusability. This could be attributed with the poor catalyst reusability as well as the difficulties in separating the catalyst powder after its use in the oxidation processes. Thus, the current work is dealing with the catalyst recover for reuse facilities to verify its sustainability.

**Fig. 15:**
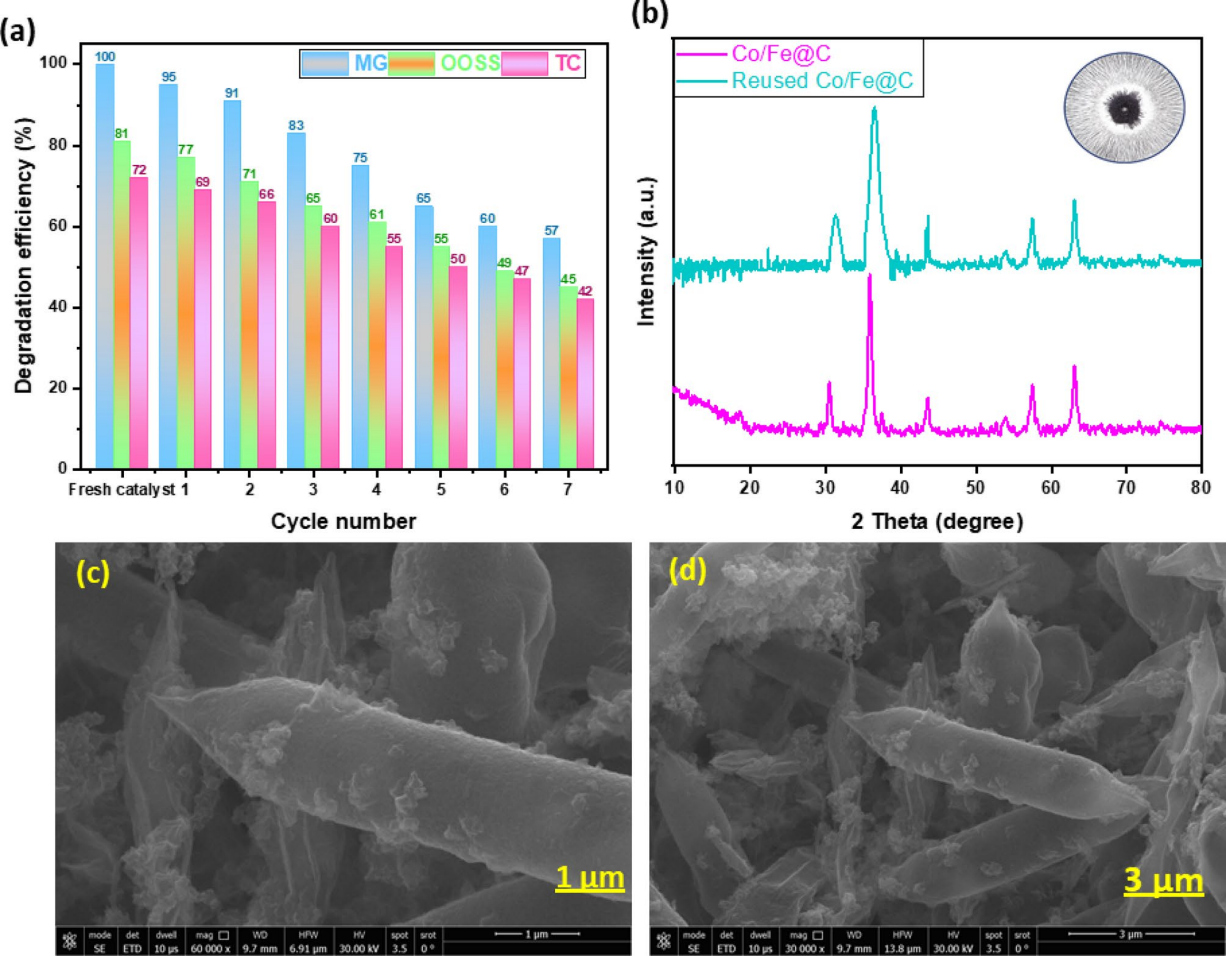
Reusability performance of Co/Fe@C (**a**) in pollutant catalytic oxidation as a function of cyclic use (**b**) XRD pattern and (**c** and **d**) SEM images of the used Co/Fe@C material

In this regard, the catalyst was collected and separated from the reaction mixture via the use of external magnetic separation process for the object of catalyst reusability. Such technique is signified as simple, efficient, evades catalyst losses and economic since it avoids electricity use. A neodymium permanent magnet (grade N42, surface field ~ 0.4 T) was placed adjacent to the reaction vessel, allowing complete collection of the magnetic catalyst within around 30–60 s. The recovered catalyst was then exposed for washing with distilled water and ethanol then dried prior to reuse for 2 h (at 100 °C). The data of catalyst reusability is exhibited in Fig. 15. As displayed in the Figure, the lessening of Co/Fe@C activity through the successive cycles was changed from 100 to 57%, 81 to 45% and from 72 to 42% for MF, OOSS and TC, respectively during the reaction time. This could be attributed by the pollutants’ molecules occupied the Co/Fe@C composite active sites and hence hinders its oxidation activity. But it is noteworthy to mention that even through the catalyst is used for several times reached to seven cycles of use that signifies that the catalyst is still possess a significant activity for oxidizing various pollutants.

**Table 4:** Performance of Bimetallic MOFs in photodegradation of pollutants.

**Type of bimetallic MOF (photocatalyst)**	**Source of activation/** **Light source**	**Type of Pollutant**	**Initial concentration** **(mg/L)**	**Catalyst dosage** **(g/L)**	**Reaction time** **(min)**	**Removal** **Rate/** **Degradation (%)**	**References**
**NH2-MIL-68 (In0.4Fe0.6)**	Visible light	TC	20	0.1	120	72	[66]
**Mn-MIL-53(Fe)−0.3/PMS system**	UV Lamp	TC	30	0.2	60	93.2	[67]
**Al/Fe-MOF**	Visible light	RhB	-	-	120	96	[68]
**Mn/Fe-MOF**	Visible light	RhB	3´10−5	0.005	120	92	[69]
**CuCo-MOF-7**	Oxidation	MB	0.2	0.005	30	100	[70]
**Cd/Zr-MOF**	Sun light	RhB	20	0.001	105	95.82	[71]
**FeCo-MOF-74**	UV Lamp	PHE	1	0.005	30	Nearly 100	[70]
**MIL 53(Fe–Cu)**	Visible light	CIP	30	1	150	57.88	[72]
**La-Y-PTC**	Mercury Lamp	MB	10	0.05	180	69.57	[73]
		MO				93.63	
**Co/Ni-MOF@BiOI**	Visible light	MB	10	0.16	240	99	[74]
**Mn-Al-MOF**	Visible light	D-Red23	80	0.005	70	84.9–100	[75]
**Zn/Co** **‑** **ZIF-8**	Visible light	MB	10	0.001	180	45	[76]
**Ni/Cu-MOF@BiOI-15**	Sun light	MB	10	1.2	<5	95	[77]
**MIL-88B**	Adsorption	OTC	50	0.125	60	90	[78]
**Co/Fe@C**	Dark	MG	10	0.004	35	100	Current investigation
		OOSS	100	0.001	10	83.38	
		TC	50	0.004	20	72.12	

*MG: Malachite green, Rh B: Rhodamine B, MB: Methylene blue, PHE: Phenanthrene, CIP: Ciprofloxacin, MO: Methyl orange, D-Red23: Direct Red 23, OTC: oxytetracycline, OOSS: Oil orange SS, TC: Tetracycline.

In parallel, after wastewater treatment, the used Co/Fe@C material is collected subjected to characterization thereafter, as displayed in Fig. 15 (b and c). XRD and SEM of the used Co/Fe@C catalyst are assessed to investigate the material stability. XRD pattern showed in Fig. 15 (b) displayed that the characteristic peaks are retained and remains after wastewater treatment. Also, according to the XRD spectra a slight increases is attained in the relative intensity of such peaks thus indicating the structural stability of the catalyst even though after cyclic Co/Fe@C material use. Further, this increase in peak broadening is directly reflects a decrease in crystallite size and/or an increase in lattice strain as a result of the catalytic cycles^53^.

The SEM image displayed in Fig. 15 (c and d) exhibited that the Co/Fe@C material is irregular particles. Compared to Fig. 3 (e, f and g) prior wastewater treatment, the surface of the material showed a deformation of the smooth surfaces into wrinkled rough surfaces. Such deterioration in morphology has been noticed after 7 cycles. This may be due to the severe defection conditions in the material due to the using of H_2_O_2_^26^. Such data is previously confirmed by the agreement of results displayed in literature^77,78^.

#### Radical scavenging test and oxidation mechanism

Overall, ^•^OH and HO_2_^•^ radicals signify the most quantitatively important active species in the catalytic oxidation system. Particularly, the hydroxyl (^•^OH) radical in particular is an effective oxidant for a varies pollutant molecules. In this regard, scavenging test is typically applied to investigate such radicals’ function in the oxidation reaction. In order to investigate the role of these radicals and the charge carriers in the oxidation process, the active species trapping experiment was conducted by using isopropyl alcohol (IPA) as.OH scavenger, ammonium oxalate (AO) as scavenger for holes (h^+^) as well as silver nitrates (AgNO_3_) as electrons (e⁻) scavenger that is presented throughout the oxidation reaction of OOSS, MG and TC^74,79^.

**Fig. 16:**
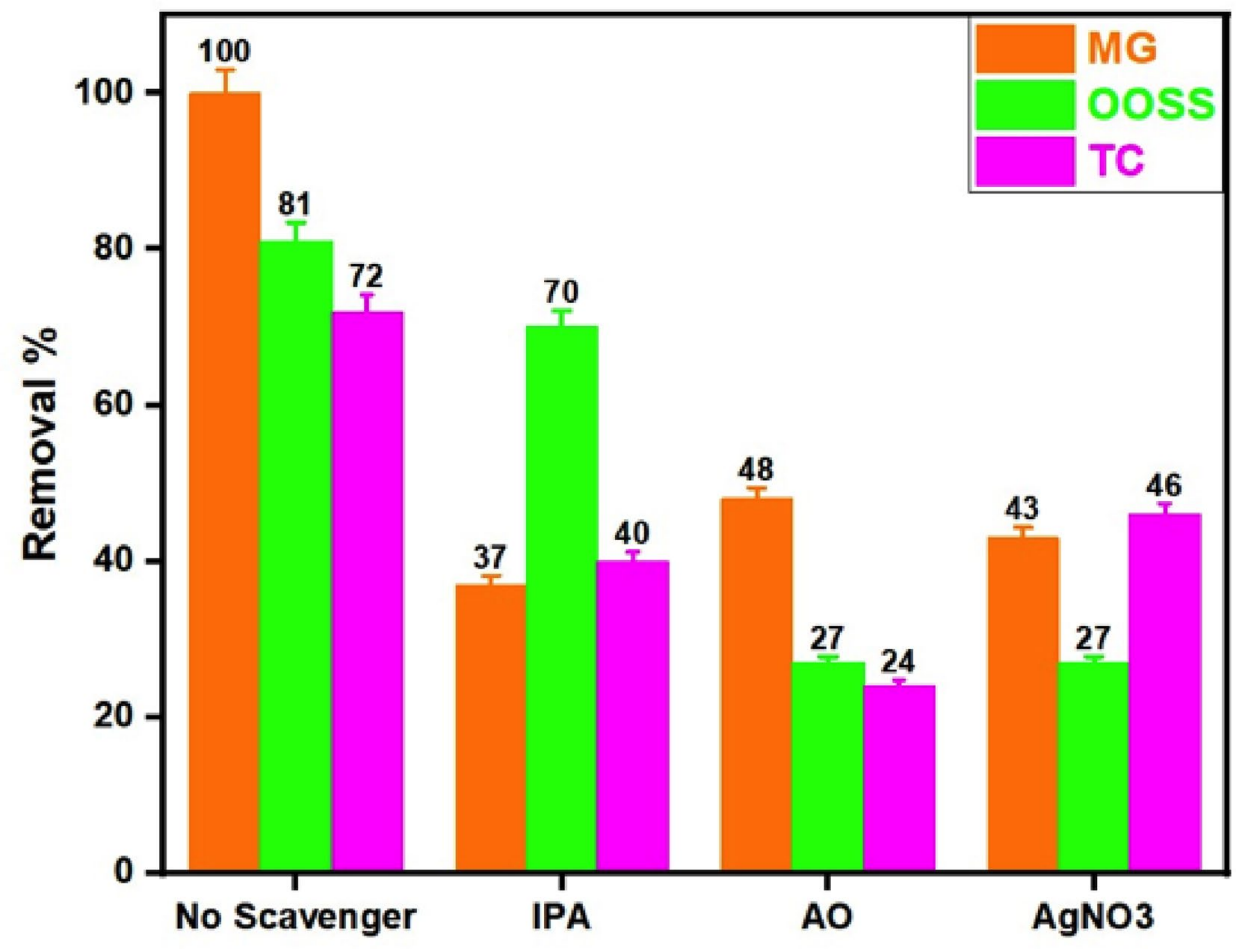
Scavenger’s series effect on the oxidation efficiency of various pollutants

Figure 16 represents the scavenger test at the optimal operating conditions of the Fenton-like reaction^80^. As shown in Fig. 16, the addition of IPA results in a reduction in the MG oxidation performance to suddenly drop from 100 to 37%. This supports the concept of the that the importance of the availability of ^•^OH in the oxidation reaction. However, a lower effect in comparable with the effect of OH radical trapping is attained when both electrons (e⁻) scavenger or holes (h^+^) scavenger are applied in the oxidation MG since the reduction in the oxidation is declined from 100% to 43 and 48%, respectively^81,82^.

The addition of AO on both OOSS and TC oxidation medium results in a decline in the oxidation efficiency from 83 to 27% and 72 to 24%, respectively. Based on such data, the results confirm the effective occurrence of holes in the oxidation reaction of both OOSS and TC. But, OOSS and TC oxidation and removal efficiency is minor affected by IPA and AgNO_3_. The oxidation efficiency is declined from 83 to 70% and 72 to 40%, for OOSS and TC respectively for OH radical trapping test. Also, the efficiency is decreased from 83 to 28% and 72 to 46%, for OOSS and TC respectively for e^−^ trapping test. Thus, such result is suggesting that e^−^ and •OH are not the primary active species for the oxidation reaction for both OOSS and TC. Hence, the sequence in which the active species’ catalytic oxidation impact on Co/Fe@C reduction could be described according to the following:

•OH > *e*^*−*^ > *h*^*+*^ for MG,

*h*^*+*^ > *e*^*−*^ > •OH for OOSS and,

*h*^*+*^ > •OH > *e*^*−*^for TC.

Commonly, Fenton/Fenton-like oxidation reaction systems generate reactive radicals from H_2_O_2_ activation through which ⋅OH radicals become the main oxidative species for pollutant breakdown. As a result, pollutants are mostly eliminated. The unsaturated metal sites of the Co/Fe@C MOFs can operate as Lewis’s acid catalysts, interacting with water molecules to produce metal hydroxides (M OH) or metal hydrates (M OH_2_). The high cationic properties of metal centres polarize water molecule polarization, which leads to their auto-deprotonation reaction^26^. The activity of Co/Fe@C depends on its unsaturated metal centres that remain after pyrolysis because these centres react with H_2_O_2_ to generate reactive oxygen species (ROS). Equation (2) through (5) illustrate the role of M^2+^ and H_2_O_2_ combination to produce M^3+^ and how M^3+^ is also converted back to M^2+^. Such metal valence states could be referred as MII and MIII whereas M signifies the metal.

2 $$\:{Fe}^{2+}+{H}_{2}{O}_{2}\to\:\:{Fe}^{3+}+{OH}^{\bullet\:}+OH\:\:\:\:\:\:\:\:\:\:\:\:\:\:\:\:\:\:\:\:\:\:\:\:\:$$

3 $$\:{Fe}^{3+}+{H}_{2}{O}_{2}\to\:\:{Fe}^{2+}+{{HO}_{2}}^{\bullet\:}+{H}^{+}\:\:\:\:\:\:\:\:\:\:\:\:\:\:\:\:\:\:\:\:\:\:\:\:$$

4 $$\:{Co}^{2+}+{H}_{2}{O}_{2}\to\:\:{Co}^{3+}+{OH}^{\bullet\:}+OH\:\:\:\:\:\:\:\:\:\:\:\:\:\:\:\:\:\:\:\:\:\:\:\:\:$$

5 $$\:{Co}^{3+}+{H}_{2}{O}_{2}\to\:\:{Co}^{2+}+{{HO}_{2}}^{\bullet\:}+{H}^{+}\:\:\:\:\:\:\:\:\:\:\:\:\:\:\:\:\:\:\:\:\:\:\:\:$$

According to Eq. (2), Fe^2+^ is converted to Fe^3+^. Furthermore, the reduction of Fe^3+^ to Fe^2+^ is occurred (Eq. 3). It is noteworthy to mention that such reaction is signified as much slower reaction in comparable with the forward reaction of Fe^2+^ with H_2_O_2_, which is considered the rate-limiting step in the homogeneous Fenton reaction where Fe^3+^ cannot be easily recovered and regenerated^34^. But, in the heterogeneous current reaction it is easier to recover and regenerated. According to the XPS results that exhibited 9% of the Fe^2+^ in the pristine Co/Fe@C changed to 20% after one cycle of the Fenton-like reaction that verifies the above-mentioned phenomenon. This suggests that some of the FeIII may have reacted with H_2_O_2_ and changed into the FeII state before reaching an equilibrium that maintained the amounts of Fe^3+^ and Fe^2+^ in the solid matrix. Also, after the first cycle of the Fenton-like reaction, the original Co^2+^ of 27% changed to 25%, suggesting that some of the initial CoII was also transformed to Co^3+^and may eventually achieve a specific equilibrium value that is dependent on the rates related to Eqs. (2–5). Subsequent runs’ XPS analysis reveals a consistent but marginally fluctuating M^2+^/M^3+^ ratio that corresponds to Co 2p3/2 and Fe 2p3/2^34^.

**Fig. 17:**
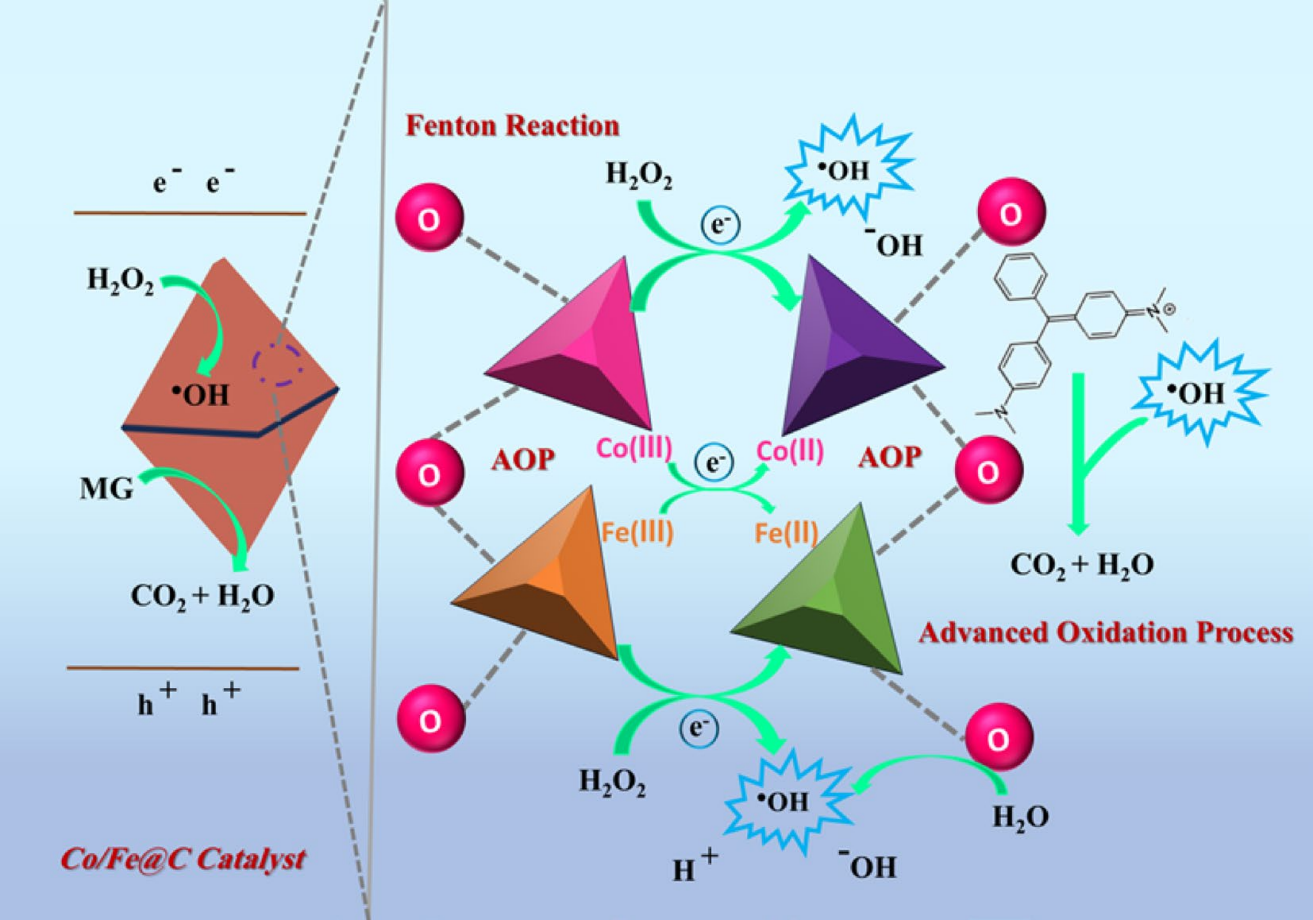
Proposed reaction mechanism of the Co/Fe@C for pollutant (MG) oxidation

The delayed oxidation of the catalyst itself or the impact of MG degraded products persisting in the Co/Fe@C could be due to the M^2+^/M^3+^ variation. Additionally, iron exists in the trivalent state as Co/Fe@C becomes formed while the synthesis uses Fe^3+^ and Co^2+^ salts, which might lead to the differences in the Co/Fe@C composition. XPS analysis (Fig. 6d and e) of the carbonized material showed Fe and Co in their Fe^3+^ and Co^2+^ states, which indicates mixed valences exist in the Co/Fe@C. Additionally, a partial distribution of Fe^2+^ and Co^3+^ states was noted, which could be explained by the quick reaction kinetics and the usage of mixed-valence salts during synthesis, which may have caused some of the metal ions to be in their energetic state. Additionally, in order to impart the metal centres mixed valent states, the mismatch sites may also be extremely reactive when they react with hydrogen peroxide to produce hydroxyl radicals and other reactive oxygen species (ROS). Thus, this could explain the high Fenton catalytic reactivity that was noted. Co/Fe@C catalyst containing unsaturated metal sites becomes possible through combining Fe^3+^ with transition metals having reduced oxidation states. The metal sites showed a good activity when moderate alkalinity medium is exists by directly transforming H_2_O_2_ into hydroxyl radicals, whereas, producing a mild acidic condition through water reaction. As a result of its quick synthesis, the Co/Fe@C has a loosely packed structure that confines MG molecules to the site of the ⋅OH radicals produced by those metal centres and aids in their destruction. Figure 17 presents a schematic mechanism for the Fenton-like degradation by the Co/Fe@C/H_2_O_2_ system, which is based on all the above-mentioned data.

#### Pollutant degradation mechanistic pathway

In typical advanced oxidation systems, malachite green (MG) meets an initial ^•^OH radical attack on the basic triphenylmethane carbon, breaking chromophoric conjugation and producing aromatic fragments such as benzoic acid derivatives and phenols. The intermediates are oxidized through electron transfer and hole-driven processes, eventually mineralizing into CO_2_ and H_2_O. Additionally, scavenger investigations consistently demonstrate that ^•^OH radicals are the main oxidants. Therefore, the synergistic action of these discovered active species, with ^•^OH playing the most important role, promotes the progressive breakdown of MG from initial oxidation, demethylation, ring opening, and thereby mineralizing them into simpler inorganic compounds^83^.

## Conclusion

To satisfy sustainability, novel modified superparamagnetic Co/Fe@C catalyst resulted from thermal shock treatment MIL-88B (Fe/Co) was conducted as an efficient treatment method for various organic pollutants mineralization. The experimental data revealed a removal efficiency that could be reached to a complete removal for dye contaminating wastewater. A short oxidation time (30 min) is recorded under dark conditions. Furthermore, the Co/Fe@C catalyst reusability showed the effectiveness of the catalyst for cyclic uses with a significant stability. Hence such introduced Co/Fe@C material revealed promising features for water remediation applications. Additionally, the reaction mechanism exhibited the effective active species in the reaction that is signified as holes or may be hydroxyl radicals. The kinetic data showed the reaction is best fitted to the second-kinetic order that describing the system. However, excess work is essential to evaluate this facility in treating real wastewater samples with different contaminants that is not handled through the current work.

## Data Availability

The data that support the findings of this study are available from the corresponding author upon reasonable request.
